# Genomic Underpinnings of Cytoplasmic Incompatibility: CIF Gene-Neighborhood Diversification Through Extensive Lateral Transfers and Recombination in *Wolbachia*

**DOI:** 10.1093/gbe/evae171

**Published:** 2024-08-06

**Authors:** Yongjun Tan, L Aravind, Dapeng Zhang

**Affiliations:** Department of Biology, College of Arts and Sciences, Saint Louis University, St. Louis, MO 63103, USA; National Center for Biotechnology Information, National Library of Medicine, National Institutes of Health, Bethesda, MD 20894, USA; Department of Biology, College of Arts and Sciences, Saint Louis University, St. Louis, MO 63103, USA; Program of Bioinformatics and Computational Biology, College of Arts and Sciences, Saint Louis University, St. Louis, MO 63103, USA

**Keywords:** *Wolbachia*, cytoplasmic incompatibility, CIF loci, evolutionary dynamics, classification, recombination, lateral transfer

## Abstract

Cytoplasmic incompatibility (CI), a non-Mendelian genetic phenomenon, involves the manipulation of host reproduction by *Wolbachia*, a maternally transmitted alphaproteobacterium. The underlying mechanism is centered around the CI Factor (CIF) system governed by two genes, *cifA* and *cifB*, where *cifB* induces embryonic lethality, and *cifA* counteracts it. Recent investigations have unveiled intriguing facets of this system, including diverse cifB variants, prophage association in specific strains, copy number variation, and rapid component divergence, hinting at a complex evolutionary history. We utilized comparative genomics to systematically classify CIF systems, analyze their locus structure and domain architectures, and reconstruct their diversification and evolutionary trajectories. Our new classification identifies ten distinct CIF types, featuring not just versions present in *Wolbachia*, but also other intracellular bacteria, and eukaryotic hosts. Significantly, our analysis of CIF loci reveals remarkable variability in gene composition and organization, encompassing an array of diverse endonucleases, variable toxin domains, deubiquitinating peptidases (DUBs), prophages, and transposons. We present compelling evidence that the components within the loci have been diversifying their sequences and domain architectures through extensive, independent lateral transfers and interlocus recombination involving gene conversion. The association with diverse transposons and prophages, coupled with selective pressures from host immunity, likely underpins the emergence of CIF loci as recombination hotspots. Our investigation also posits the origin of CifB-REase domains from mobile elements akin to CR (Crinkler-RHS-type) effectors and *Tribolium* Medea1 factor, which is linked to another non-Mendelian genetic phenomenon. This comprehensive genomic analysis offers novel insights into the molecular evolution and genomic foundations of *Wolbachia*-mediated host reproductive control.

**Significance**

The endoparasitic bacterium *Wolbachia* manipulates host reproduction via cytoplasmic incompatibility (CI) resulting in non-Mendelian offspring survivorship. The CI primarily depends on a two-gene system, *cifA* and *cifB*, which features polymorphic domain architectures, multiple effector functions, genomic copy number variation, and rapid diversification between closely related strains. Our comprehensive comparative genomics analysis of the CIF loci establishes a new classification of CIF systems and uncovers remarkable variability in the gene composition and organization of these loci. We demonstrate that the observed domain architectural and sequence diversity of the genes in the CIF loci are attributable to extensive gene transfers and interlocus recombination facilitated by associated prophages and transposons. We present evidence that a consistent set of multiple genes associated with the CIF loci, including diverse endonucleases, deubiquitinating peptidases (DUBs), and a membrane-lipids-targeting toxin are potential effectors that might augment the cifA/cifB core in manipulating host biology. Our findings suggest that the CIF loci serve as recombination hot spots, leading to diversification at multiple levels, including genomic organization, constituent gene families, and protein domain architectures.

## Introduction

*Wolbachia* is a genus of obligate endosymbiotic Gram-negative alphaproteobacteria found in a wide range of arthropods and nematodes (Hilgenboecker et al. 2008; Werren et al. 2008), including some that are human parasites and disease vectors (Lefoulon et al. 2016; Sinha et al. 2019; Adams et al. 2021). Studies estimate that over 50% of arthropod and nematode species carry *Wolbachia* (Hilgenboecker et al. 2008). These bacteria mainly inhabit host reproductive organs and are transmitted from mothers to offspring (Frydman et al. 2006; Zug and Hammerstein 2012). Extensive research has demonstrated that *Wolbachia* can manipulate insect reproduction, inducing phenomena such as feminization, parthenogenesis, male killing, and sperm–egg incompatibility (Werren et al. 2008). Cytoplasmic incompatibility (CI) is a prominent non-Mendelian genetic phenomenon that results in embryonic mortality in the offspring of mating between animals of the same species that differ in their status of *Wolbachia* infection. It might express either unidirectionally or bidirectionally: the former case involves the mating of *Wolbachia*-infected males with uninfected females and the consequent mortality of their embryos (Laven 1967; Yen and Barr 1971). The bidirectional version involves the mortality of the embryos arising from the mating between a male and a female, each infected by a distinct strain of *Wolbachia* (Atyame et al. 2014). However, reciprocal matings between an infected female and an uninfected male or between two animals where both sexes are infected by the same *Wolbachia* strain are compatible (Serbus et al. 2008; Beckmann et al. 2017; LePage et al. 2017). Thus, CI acts as a driver for increasing the presence of an uniparentally inherited symbiont in the population (Field et al. 1999; Hancock et al. 2011a, 2011b; Shropshire et al. 2018).

Recent studies have implicated a system comprised of two adjacent genes (*cifA* and *cifB*; Beckmann and Fallon 2013; LePage et al. 2017) in *Wolbachia* as the determinants of CI in their *Drosophila* host (Beckmann et al. 2017; LePage et al. 2017; Shropshire et al. 2018; Chen et al. 2019; Hochstrasser 2023). Studies on the *cifB* genes revealed that they code for proteins with variable domain compositions and architectures, such as CinB (CI-inducing nuclease) containing two catalytically active and two inactive domains displaying the restriction endonuclease (REase) fold (Beckmann et al. 2017; Chen et al. 2019), and CidB (CI-inducing DUB), harboring apparently inactive REase domains coupled to a C-terminal papain-like peptidase domain related to the de-SUMOylating enzyme Ulp1/SMT4 (Beckmann et al. 2017; LePage et al. 2017). Despite being closely related to the SUMO-processing peptidase, this Ulp1/SMT4 domain has been shown to act on ubiquitin conjugates (Stephens et al. 1998; Beckmann et al. 2017). Multiple studies have pointed to the sufficiency of the CifB products for inducing CI (Sun et al. 2022). CifA products, namely CinA and CidA, have a set of six HEAT repeats that might be involved in protein–protein interaction (Adams et al. 2021; Xiao et al. 2021; Wang et al. 2022). A double transgenic expression of *cifA* and *cifB* in male *Drosophila* causes CI when mating with an uninfected female, which can be rescued by mating with an infected female or overexpressing *cifA* in uninfected females. Thus, CifA was proposed to function as the rescue (or antidote) component in this CI system (Beckmann et al. 2017; LePage et al. 2017; Shropshire et al. 2018). This led to the development of the toxin–antidote (TA) model for CI (Beckmann et al. 2017, 2019; Terretaz et al. 2023). However, several other models have been proposed (Poinsot et al. 2003; Namias et al. 2022), including the host-modification (H-M) model wherein the effector can modify the host sperm function, and two-by-one model wherein both CifA and CifB are seen to be necessary components for mediating the lethality that characterizes CI (LePage et al. 2017; Shropshire and Bordenstein 2019; Shropshire et al. 2019).

In addition to the diversity of biochemical functions exhibited by both CifB and CifA components, recent research has unveiled several intriguing aspects regarding the evolution and genomic features of the system. First, even among different strains of the same *Wolbachia* species, the number of genomic copies of the CIF system varies (Bonneau et al. 2018b; Martinez et al. 2021). These distinct CIF copies are classified into four or five types (Lindsey et al. 2018; Martinez et al. 2021; Quek et al. 2022) and their interactions are believed to underlie the bidirectional CI patterns observed between the individuals infected by distinct *Wolbachia* strains (Atyame et al. 2014). Furthermore, the rapid diversification of both CifB and CifA through recombination has been described (Bonneau et al. 2018a). While the expansion and diversification of CIF could be attributed to prophages associated with CIF operons (LePage et al. 2017), a recent study revealed that some *Wolbachia* in *Anopheles* carry CIF systems with no associated prophage elements (Quek et al. 2022), indicating that additional mechanisms may exist to facilitate recombination/mobility in CIF systems. Given the observed diversity in biochemical functions and unique features of the genomic organization of the CIF system, our study aimed to explore its evolutionary trajectories through comparative genomics, gene neighborhood, phylogenetic, and sequence–structure analyses.

As a result, we have established a new classification of CIF systems, expanding the previously defined five types into ten types. We discovered remarkable variability in gene composition and organization of these loci, and we demonstrated that diversification involves extensive independent gene transfers and interlocus recombination, potentially facilitated by associated prophages and transposons. Additionally, we identified several potential effectors on the CIF loci, including diverse endonucleases, deubiquitinating peptidases (DUBs), and a toxin-targeting membrane lipid. Our discoveries indicate that the CIF loci function as areas of high recombination, fostering diversification across multiple levels, such as genomic organization, gene-family-composition, and protein domain architectures.

## Materials and Methods

### Protein Sequence and Structure Analyses

The PSI-BLAST program (Altschul et al. 1997), an iterative sequence profile search program, was used to collect CifB homologs by searching against the NCBI nr protein database. To accurately identify true homologs of CifB, we scrutinized protein hits in each iteration based on two additional criteria. First, we examined the domain architecture, focusing on the presence of four tandem REase domains characteristic of CifB proteins. Second, we assessed the upstream gene to verify its identity as CifA. The searches also included CR-effectors (Zhang et al. 2016); however, these could be easily distinguished from *bona fide* CifB proteins by virtue of their diverse domain architectures and operonic associations. Setting an *e*-value inclusion threshold of 0.0001 for significance, we successfully retrieved the majority of CifB homologs while minimizing the inclusion of CR-effectors. Subsequent analysis of the domain architecture revealed that most proteins exhibited the typical CifB structure, characterized by a 4-REase unit architecture. In contrast, CR-REase proteins typically showcased a conserved domain architecture, featuring a leading ATPase domain followed by a REase domain.

In addition, we uncovered some sequences annotated as insect proteins that showed close relationship to *Wolbachia* sequences. To explore the possibility of incorrect annotation or assembly, we employed two strategies. First, we conducted BLASTP searches using these eukaryotic protein candidates, and subsequently examined the species distribution of their closest homologs. Our rationale was that if we identified many close homologs from related eukaryotes, the query protein would likely be a true eukaryotic sequence. Conversely, if its closest homologs were primarily bacterial, its eukaryotic origin would be less likely. Second, we analyzed the gene neighborhood of these candidates. If the neighboring genes were also from eukaryotes (specifically, arthropods or nematodes), we retained them. However, if they were bacterial, we removed them. All data presented in this study has been cleaned using these two methods. These analyses enabled us to confidently retrieve 205 unique CifB protein sequences from the nr database, corresponding to 295 occurrences across 149 genomes in GenBank. Furthermore, leveraging the curated CifB collection, we retrieved the associated CifA proteins by extracting the direct upstream gene of each cifB gene. We utilized the edirect programs (https://www.ncbi.nlm.nih.gov/books/NBK179288/) to extract the corresponding genomes and taxonomy information. Notably, among 154 *Wolbachia* genome records with protein annotations, 115 genomes contained CIF homologs. It is worth noting that several CIF homologs share the same NCBI nr accession numbers within these 115 *Wolbachia* genomes.

Multiple sequence alignments (MSAs) were constructed using the PROMALS3D program, which integrates hidden Markov model (HMM) comparisons and secondary structural alignment, consistently yielding improved alignments (Pei et al. 2008). Profile–profile searches were performed using the HHpred program (Steinegger et al. 2019). The conservation pattern of the MSA was calculated using a consensus method based on different categories of amino acid physicochemical properties developed by Taylor (1986). The consensus was determined by examining each column of the MSA to determine if a threshold fraction (either 75% or 80%) of the amino acids belongs to a defined category. The MSAs for the major protein families within the CIF loci can be found in the supplementary data, Supplementary Material online.

For predicting the tertiary structure of the proteins, the AlphaFold2 program (Jumper et al. 2021) was utilized, and models with the highest predicted Local Distance Difference Test scores were selected. The determination of domain boundaries of proteins was guided by both sequence conservation patterns and the PAE matrix provided by AlphaFold2 (Jumper et al. 2021). Structural comparisons were conducted using the DALI program (Holm et al. 2023). Structure analysis and visualization were carried out using the molecular visualization program PyMOL (DeLano 2002).

### Gene Neighborhood Analysis

We employed gene neighborhood analysis to identify conserved genes in the cifA–cifB loci to gain insights into the molecular mechanisms of CI. The upstream and downstream genes (15 genes on each side of the query) of collected *cifB* genes were retrieved from the NCBI GenBank file. Both *cifB* genes and their neighboring genes were clustered using the similarity-based clustering method, BLASTCLUST, which relies on BLAST scores for single-linkage clustering (https://ftp.ncbi.nih.gov/blast/documents/blastclust.html). Each cluster and its corresponding proteins were annotated with their domain architectures using the HMMSCAN program (Steinegger et al. 2019), which was run against the Pfam database (Mistry et al. 2021), as well as our curated domain profiles. To determine conserved gene neighbors, we applied three criteria: (i) proximity to CifB/A genes on the genome; (ii) persistence of these associations across at least two distinct CIF types; and (iii) dissimilarity or lack of high similarity, suggesting a history of diversification.

To assess the statistical significance of the enrichment of particular genes in the neighborhood of the *cifA–cifB* gene dyad, complete *Wolbachia* genomes were surveyed and the genes belonging to the six functional classes mentioned above, along with the core dyad itself were numerically coded. Similarly, the remaining genes in the genome were then numerically coded as “0”; 10^6^ simulated genomes were created from these real genomes by random rearrangements typical of genome reorganization. These were then queried to determine the number of cases of comparable enrichment of the CIF-associated genes in at least one similarly sized locus as in the real genomes and compute a *P*-value from the same. The simulations were performed using the R-language.

### Molecular Phylogenetic Analysis

We utilized various phylogenetic analysis methods, including ML analysis using PhyML (Guindon et al. 2010), BI with BEAST (Suchard et al. 2018), NJ analysis conducted with MEGA7 (Kumar et al. 2016), a rapid approximately ML inference using FastTree (Price et al. 2009), and network tree analysis utilizing SplitsTree (Huson and Bryant 2006), to reconstruct the phylogenetic relationships of proteins in this study.

The phylogenetic relationships of CifA and CifB proteins were established using five distinct computational methods: ML estimation with PhyML, BI with BEAST, NJ algorithm in MEGA-NJ, FastTree, and Network-based SplitsTree analyses. The PhyML approach was implemented using the JTT (Jones-Taylor-Thornton) substitution model (Jones et al. 1992) integrated with a discrete Gamma distribution (four rate categories), allowing for heterogeneity in evolutionary rates across different sites. This method prioritized the tree topology exhibiting the maximal log-likelihood value. In the BEAST-BI framework, a JTT model coupled with a similar Gamma distribution (four rate categories) was employed. This was augmented by executing dual Markov chain Monte Carlo (MCMC) simulations, each encompassing 1 million states and recording data at intervals of 10,000 steps. The convergence and stability of these MCMC simulations were assessed using the Tracer 1.7.1 software, with the initial 10% and 27% of the iterations discarded as burn-in for CifA and CifB, respectively. The MEGA-NJ analysis also utilized the JTT model with a four-category Gamma distribution to account for site rate variability and included a bootstrap test with 100 replicates for robustness assessment. The FastTree and SplitsTree algorithms were executed using their default settings. To validate the phylogenetic clades obtained, several statistical support metrics were employed: approximate likelihood-ratio test (aLRT) SH-like values from PhyML, Bayesian posterior probabilities from BEAST-BI, bootstrap values from MEGA-NJ, and SH-like local support values from FastTree (supplementary figs. S1 and S2, Supplementary Material online).

To infer the evolutionary relationship of Ulp1/SMT4, OTU, and PL-OTU, we conducted PhyML analysis using different substitution models. The CpREV model was applied for the Ulp1/SMT4 family, while the JTT model was used for both OTU and PL-OTU families. Initial trees for the heuristic search were obtained using the Neighbor-Join and BioNJ algorithms, and a discrete gamma distribution (four categories) was applied in all three trees to account for rate heterogeneity among sites. aLRT SH-like analysis was performed to evaluate the significance of the phylogenetic groupings.

For investigating the recombination pattern within type I CifB sequences, PhyML analysis was applied to the CifB four REase domains and Ulp1/SMT4 domain separately. The JTT model with a discrete gamma distribution (four categories) was used to model rate heterogeneity among sites. Initial trees for the heuristic search were obtained using the Neighbor-Join and BioNJ algorithms based on pairwise distances estimated using the maximum composite likelihood approach. Bootstrap analysis with 100 repetitions was conducted to evaluate the supporting values.

The resulting trees with the highest log likelihood from PhyML analysis were visualized using the MEGA7 program. Branch lengths were measured in substitutions per site, and supporting values were displayed next to the major branches.

Finally, phylogenetic network analysis was performed to assess inconsistent evolutionary histories among different regions of the protein components prevalent in CIF loci, potentially caused by recombination and/or gene transfers. The network tree was constructed using the Neighbor-net method in the SplitsTree program, based on uncorrected *P* distances.

## Results

### The Phyletic Spread and Copy Number Variation of CIF Systems

**Fig. 1.**
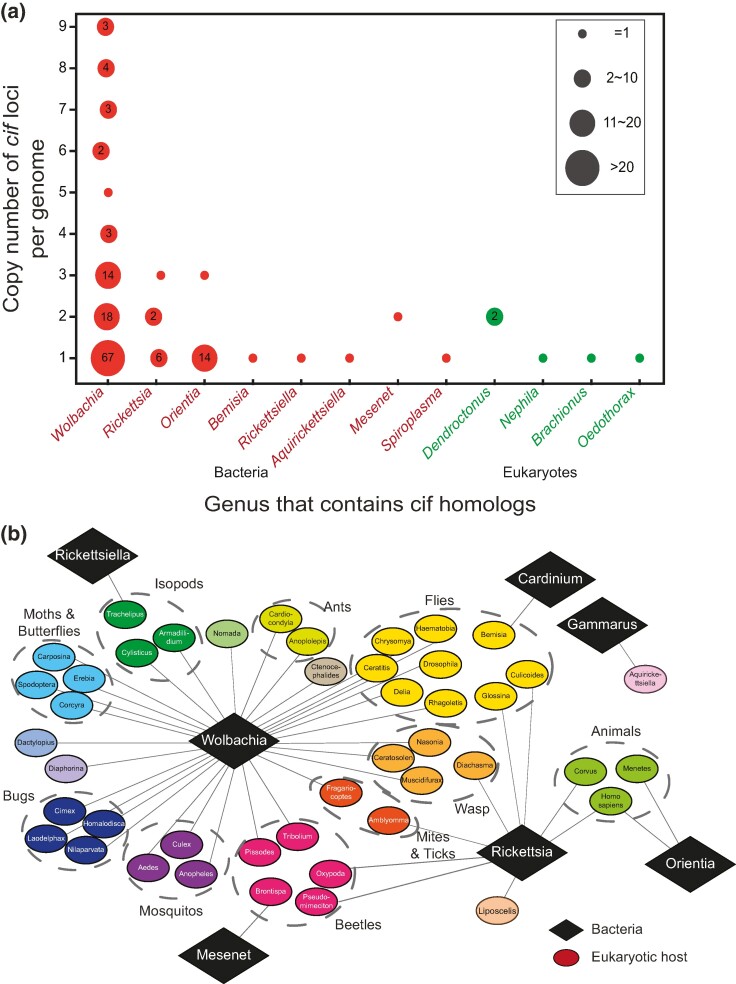
a) The distribution of CifB components across different phyla and the variations in their copy numbers within genomes. The size of each circle corresponds to the number of genomes that contain the specified copies of CIF homologs. b) Distribution of eukaryotic hosts of the intracellular bacteria containing CIF homologs. The bacteria are represented by black diamonds, while their hosts are depicted as ellipses colored based on their categories, which include isopods, ants, flies, wasps, ticks, beetles, mosquitoes, bugs, moths, butterflies, and others.

We first used the CifB protein (WP_038228284.1; LePage et al. 2017) as a query to search against the NCBI nonredundant (nr) protein database using position-specific iterated BLAST (PSI-BLAST) with a significance threshold of 0.0001. We retrieved 205 unique protein sequences from the nr database, corresponding to 295 occurrences across 149 genomes in GenBank. While the majority of these homologs were from *Wolbachia* (115 out of 154 annotated *Wolbachia* genomes in GenBank), we also recovered versions from other intracellular bacteria such as *Rickettsia*, *Orientia*, and several eukaryotic genomes (Fig. 1a). This is keeping with the previously reported integration of the *Wolbachia* genome into host chromosomes (Nikoh et al. 2008). The hosts of the *Wolbachia* and the remaining intracellular bacteria recovered in these searches included several arthropods and other animals (Fig. 1b). Importantly, when we mapped the identified CifB sequences to their individual genomes, we observed large variability in the copy number of the CifB genes between the genomes (Fig. 1a). This variation is mostly found in *Wolbachia*, in which 67 genomes contain one copy while 48 genomes contain two to nine copies of CifB genes (Fig. 1a). A similar pattern was found for CifA, although in certain genomes, CifB is apparently not accompanied by a CifA homolog (see below). This suggests that the CIF system shows a certain tendency for proliferation in the otherwise highly reduced *Wolbachia* genomes.

### Classification of the CIF Systems Reveals Ten Distinct Types

**Fig. 2.**
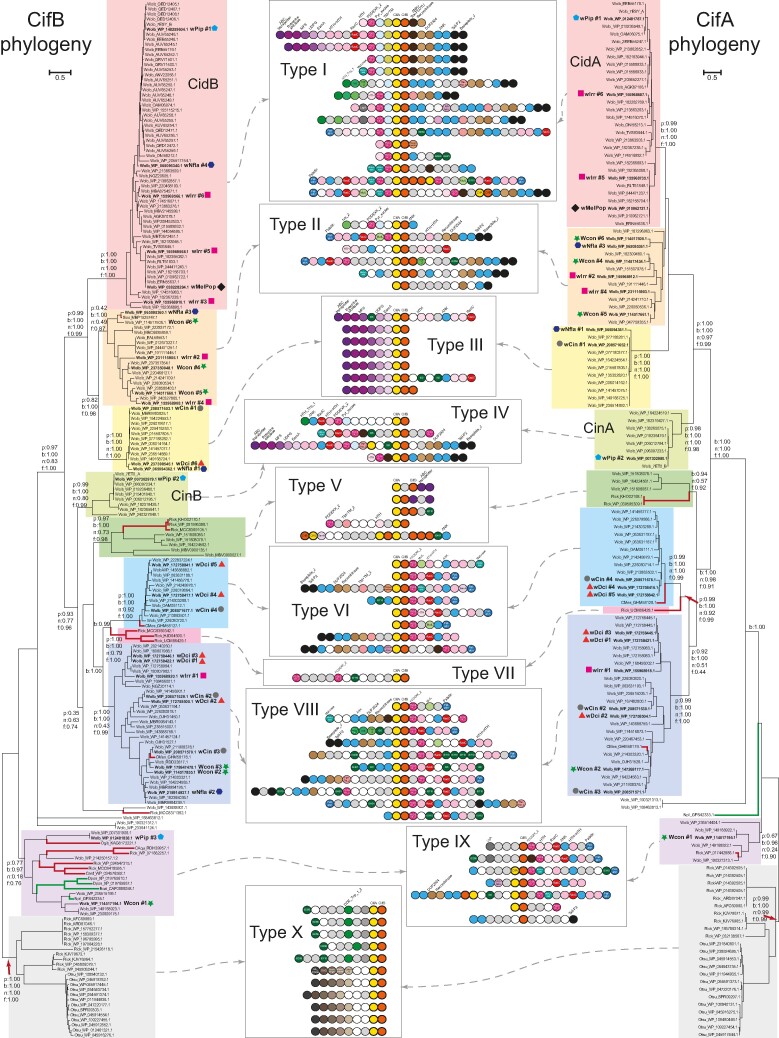
Classification of the CifB (left) and CifA (right) proteins and the representative gene neighborhoods for each major clade. The ML phylogeny with the highest log likelihood, inferred based on the JTT model, is shown with the supporting values for the major branches from three phylogenetic methods: *p* for PhyML aLRT SH-like value, *b* for BI posterior probability value, *n* for MEGA-NJ bootstrap value, and *f* for FastTree SH (Shimodaira-Hasegawa)-like local support value. The branches of sequences from *Wolbachia* are shown in black, whereas the sequences from other intracellular bacteria are in red, and the sequences from eukaryotes are in green. Each sequence is represented by the abbreviated species name followed by the NCBI accession number, and the selected sequences are further labeled with their species and strain information followed by the series number and representative symbol. The major ten clades are highlighted in different background colors. The representative operonic structures of CIF loci of these major clades are shown between the trees. Each circle indicates a single gene and is colored by differently based on their domain annotation, shown either on the top of each cluster of operonic structures or inside the circles. The gray and white circles present the hypothetical genes and pseudogenes without annotation, respectively. Species abbreviations used in the figures are as follows: Wolb, *Wolbachia*; Rick, Rickettsia; Sixo, *Spiroplasma ixodetis*; CMes, “*Candidatus* Mesenet longicola”; Ogib, *Oedothorax gibbosus*; CAqu, “*Candidatus* Aquirickettsiella gammari”; Card, *Cardinium*; Otsu, *Orientia tsutsugamushi*; Dpon, *Dendroctonus ponderosae*; Bcal, *Brachionus calyciflorus*; Npil, *Nephila pilipes*.

To understand the evolutionary trajectory of the CIF systems, we conducted phylogenetic analysis for both the CifB proteins and the associated CifA proteins using four independent methods, namely maximum likelihood (ML) analysis, Bayesian inference (BI), neighbor-joining (NJ) analysis, and a rapid approximately ML inference (Price et al. 2009; Guindon et al. 2010; Kumar et al. 2016; Suchard et al. 2018). Previous studies had classified the known CIF systems into four or five distinct types (Lindsey et al. 2018; Quek et al. 2022), where CidB/A belongs to type I and CinB/A belongs to IV. In contrast, based on the four different methods, our phylogenetic analysis with the newly retrieved CifB sequences consistently revealed the existence of ten distinct major clades designated as types I to X. Types I to V correspond to the previous classification, while types VI to X represent newly identified clades (Fig. 2, left; supplementary fig. S1, Supplementary Material online). Specifically, type I CifB homologs form a sister clade to the higher-order clade uniting types II and III CifB homologs. In contrast, type IV CifB homologs emerged as the basal group of a clade, uniting CifB homologs of types V, VI, VII, and VIII. Type IX CifB homologs were located outside of types I to VIII, while type X CifB was the most basal clade of all CifB homologs (Fig. 2, left). The tree of the CifA proteins, typically encoded upstream of the CifB proteins, also featured ten distinct clades (Fig. 2, right; supplementary fig. S2, Supplementary Material online). Comparing the two tree topologies, we found that the higher-order relationships between the CifA and CifB types were mostly comparable (Fig. 2), suggesting that CifA and CifB homologs had similar evolutionary trajectories—they were maintained as a dyad and expanded together via duplication events. This coevolution also implied that CifA and CifB are strongly functionally coupled. It is worth noting that a previous study (Martinez et al. 2021) proposed a recombination between CifA and CifB in several CIF types. However, their recombination signals appear to be attributable to unresolved tree relationships resulting from the inclusion of identical sequences.

Among these CIF types, clades I to VIII form a statistically well-supported higher-order clade. While the majority of the sequences from these types are from *Wolbachia*, we found strong support, for example, from other endosymbiotic/parasitic bacteria belonging to some of these clades: WP_081996388.1 (*Rickettsia felis*) in type V; GHM58178.1 (“*Candidatus* Mesenet longicola”, a CI-causing bacterium in the leaf beetle *Brontispa longissima*; Takano et al. 2021) in type VIII; some *Rickettsia* sequences in type VII. These appear to represent clear examples of the lateral exchange of the CIF loci between different endosymbiotic/parasitic bacteria. Given the preponderance of *Wolbachia* in the above clades, we tentatively propose that it might have been the source genus from which these versions spread to other bacteria. In contrast, type IX includes representatives from a wider diversity of genomes, including *Wolbachia*, various phylogenetically distant endosymbiotic bacteria, such as the gammaproteobacterium *Aquirickettsia*, which is related to *Legionella*, and *Cardinium*, the endosymbiont of the whitefly *Bemisia tabaci* from the Bacteroidetes phylum, which is related to *Amoebophilus*. These versions are also integrated into genomes of host arthropods, such as scorpions. These points to a more extensive lateral transfer between diverse organisms in this group. Type X is only present in *Rickettsia* and *Orientia.* The presence of a basal version of the CIF loci in these bacteria, together with the fact that the genus *Wolbachia* is nested within a radiation of *Rickettsia*-like bacteria (Hertig 1936; Anderson and Karr 2001), suggests that the ancestral CIF locus had emerged prior to the radiation of this clade of endosymbiotic/parasitic bacteria.

Interestingly, the consensus phylogeny revealed that in several *Wolbachia* genomes, the encoded CIF systems belonged to different types. For instance, in wPip isolated from mosquito, the three CifAB pairs belong to types I, IV, and IX; in wIrr isolated from *Haematobia irritans* (the biting horn fly), the six systems are distributed among type I (three copies), type II (two copies), and type VIII (one copy); and in wDci isolated from *Diaphorina citri* (the Asian citrus psyllid), six systems are type III (one copy), type VI (two copies), and type VIII (three copies; Fig. 2). This suggests that the multiple copies of the CIF systems encoded in a given genome were not the result of strain-specific expansion via duplication. Instead, these repertoires appear to have diversified through the accretion of multiple copies via horizontal exchange between different genomes.

### Diversity of the CIF-Associated Genomic Loci and Coupling with Various Transposons and Novel Effectors

**Fig. 3.**
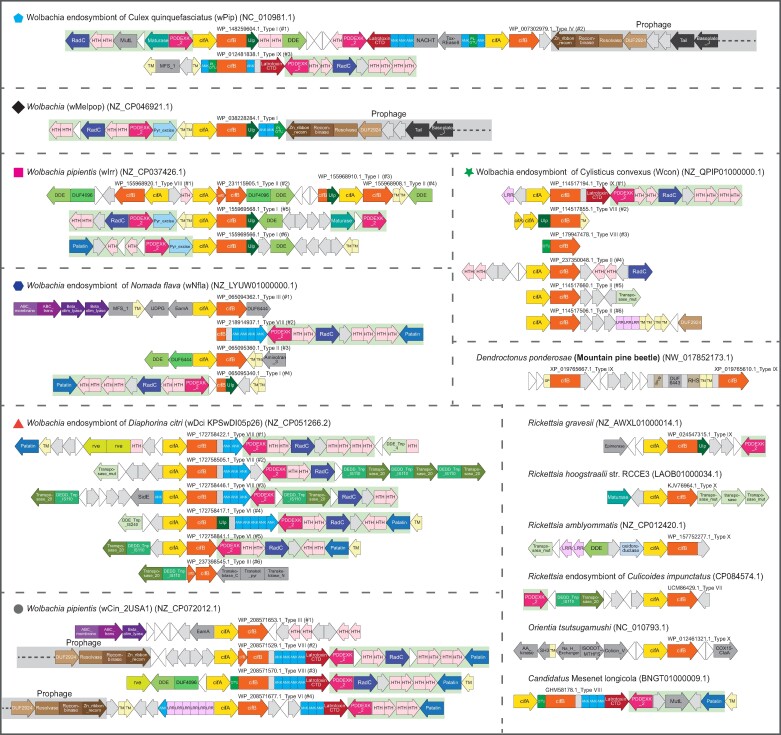
Distinct set of CIF loci and their detailed operonic structures in representative *Wolbachia* strains and other species. Each genome was labeled by the species name and strain information in brackets followed by its NCBI nucleotide accession number. Different loci of the same genome are indicated by the accession numbers of their CifB proteins and highlighted by serial numbers in bracket. The genes on the loci are shown as arrow blocks and annotated by the major domains of their encoded proteins, presented as separate rectangular segments, and highlighted in different colors. The hypothetical genes and the pseudogenes are shown as gray arrow blocks and white triangles, respectively. Selected genomes highlighted in Fig. 2 are labeled by different symbols in front of the species name.

We then investigated other mechanisms that might have led to the extensive gene transfer and accretion of the above-reported CIF types. Previous studies have reported that the CifAB operon is carried by prophages (LePage et al. 2017; Bordenstein and Bordenstein 2022), which could explain the gene transfers. However, a recent study showed that some *Wolbachia* in *Anopheles* carry CIF systems with no associated prophages (Quek et al. 2022), indicating that additional mechanisms may exist to facilitate the transfers of CIF systems. Further, their integration into the host genome also suggests that there might be additional mechanisms mediating their dispersal. To investigate this, we conducted a systematic gene neighborhood analysis to identify other genes or components that might be associated with the CIF operons. We retrieved 15 upstream and 15 downstream genes associated with the aforementioned CifAB operons. We classified them using BLASTCLUST, a sequence-similarity-based clustering method, and annotated their domain architectures through the hmmscan program, utilizing both the Pfam database and our custom profile database (details provided in the Materials and Methods section). Figure 2 shows a general picture of the curated genomic loci that contain the CIF operons, and Fig. 3 provides a detailed annotation by domains for several representatives from seven different *Wolbachia* genomes, a few other intracellular bacterial species, and a eukaryotic species.

Our analysis revealed a striking variation in both gene composition and genomic organization of CIF-containing loci. We confirmed the association of the CifAB operons with prophage elements; however, this association is limited and only seen in a subset of a given CIF type. For instance, a subgroup of type I and some from types II, IV, VI, and VIII (Figs. 2 and 3) show associations with prophages. In addition to prophages, despite significant variation in gene composition in these loci, we identified several additional genes that display associations with most CIF operons from different types. By studying their domain architectures, we can categorize them into several functional groups: (i) effectors of the Ankyrin (ANK) repeat type, either fused with CifB protein or encoded by genes adjacent to the CIF operons; (ii) multiple DUB families, including OTU, Ulp1/SMT4, PL-OTU, and C58-like (peptidase related to *Yersinia pestis* effector YopT) that are also either fused or operonically associated with CifB; (iii) various transposable elements, that include DDE (a RNase H fold nuclease) retroelements coding for a reverse transcriptase and integrase (group II intron maturase), and transposases of several families, such as IS3, IS4, IS5, IS6, IS110, IS256, IS481, and IS630; (iv) a transposon with the transposase containing a distinct REase domain (Pfam: PDDEXK_2), which carries a diversifying set of cargo genes including multiple effectors such as RadC-type JAB domain of the deaminase-like fold (Iyer et al. 2011), and pyrimidine dimer DNA glycosylase/endonuclease V (Pfam: Pyr_excise), and the Patatin-like phospholipase family protein (Burroughs et al. 2015), which is predicted to cleave the fatty acids of lipids in inner layer of the membrane bilayer; (v) many transposon-encoded DNA-binding domains, including distinct HTH domains and the RAMA DNA-binding domain (partly matching Pfam DUF2924; Iyer et al. 2016); (vi) two other elements comprising of multiple genes coupling with different types of CIF loci. For example, the “ABC transporter-EamA” element is coupled to several versions of types I, III, and IV; and another conserved element is linked to a group of type X loci (Fig. 2).

An analysis of complete *Wolbachia* genomes revealed that, on average, each such CIF locus displayed a clustering of at least seven genes from the above-described set of functional groups (in addition to the core of *cifA*/*cifB*). We ran a simulation to test if such an enrichment in the CIF loci could have emerged by chance alone. Even in the most extreme case, the genome of the *Wolbachia* endosymbiont of *D. citri*, which has at least 37 distinct genes belonging to the above six categories, and considering the longest CIF locus (19 genes) the chance association of these genes into clusters typical of the CIF loci was highly improbable (cluster with six associated genes, *P* = 0.000393; cluster with seven associated genes, *P* = 2.5e−05; cluster with eight associated genes, *P* = 1e–06). This suggests that the clusters we observe here are functionally significant associations. Nevertheless, our analysis indicated that these CIF neighborhoods from the same or different genomes, especially those in *Wolbachia*, exhibit variability in terms of the individual genes included in them and their gene neighborhood organization (Fig. 3). This supports the proposal that these loci encompassing the CIF genes have undergone lateral transfers and extensively recombined between distant genomes. More importantly, given their frequent association with prophages and various types of transposable elements (Bordenstein and Bordenstein 2022), we propose that the observed copy number variation and gene compositional diversity of CIF-associated loci are shaped by the recombinogenic capabilities of these elements. The strong association of many potential effector genes in the CIF neighborhoods suggests that they might also contribute to the interaction between *Wolbachia* and their hosts.

### Structural and Functional Diversity of CifB

**Fig. 4.**
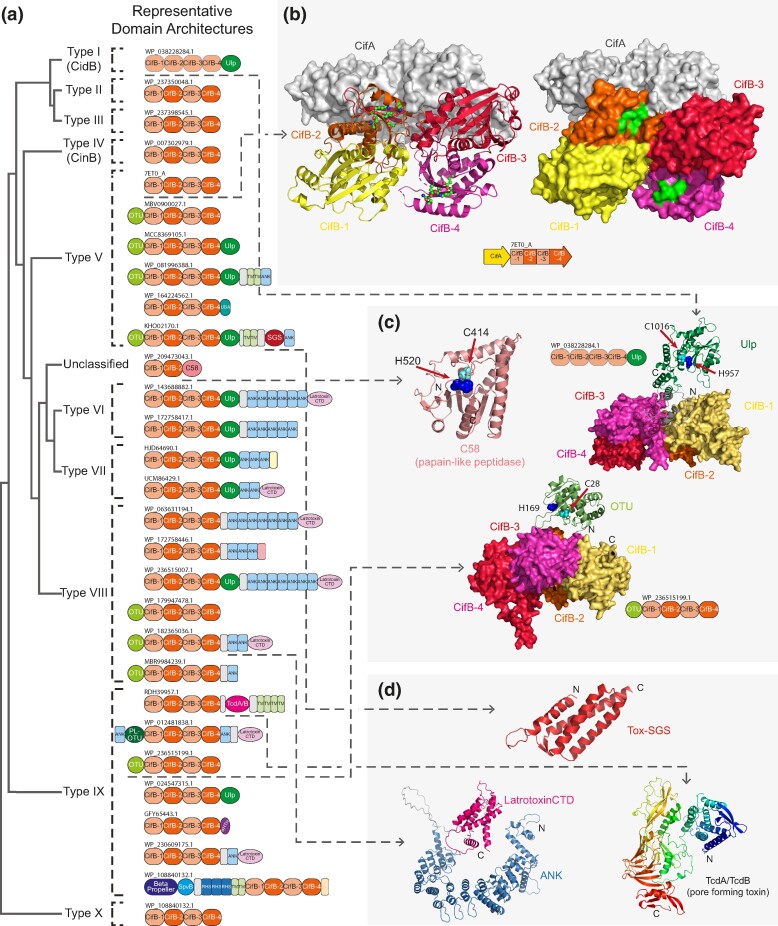
a) Representative domain architectures of the CifB proteins of each major clade. For the central four REase domains of cifB proteins, the ones that carry the conserved catalytic site residues are shown in orange, whereas those with no apparent catalytic site residues in light orange. b) Crystal structure of the CifB–CifA complex, and c) and d) AlphaFold structures of the different DUB domains, and other associated toxin domains. In the structures, the four REase domains are shown in the surface view in different colors, while other domains are shown in cartoon view, with their catalytic residues (of Ulp1/SMT4 and OTU families) labeled and highlighted (His in blue and Cys in cyan).

The toxicity of the *Wolbachia* CI system is primarily determined by the CifB component (Beckmann et al. 2017; LePage et al. 2017; Shropshire et al. 2018; Chen et al. 2019). However, prior studies have attributed this toxicity to different domains in the protein, viz., the Ulp1/SMT4 peptidase domain in type I CifB (CidB) or the REase domains in type IV CifB (CinB; Beckmann et al. 2017; LePage et al. 2017). To better understand this situation, we systematically investigated the domain architectures and structures of the CifB components across all ten types identified in this study (Fig. 4). We first performed a domain architecture analysis on all CifB proteins, selecting representatives for each type based on dominant domain architectures. Subsequently, we modeled the structures of chosen representatives using AlphaFold2. Through structural similarity searches with the DALI program (Holm et al., 2023), we identified a common core structure shared among all models. This core structure was then dissected into four domains using profile–profile searches and predicted aligned error (PAE) matrices generated by AlphaFold2, guided by the principle that individual domains typically exhibit a globular structure, which folds independently. For each domain, DALI searches were conducted to identify structural homologs consistently revealing a REase fold. Further analysis of sequence conservation patterns for each domain enabled the identification of potential catalytic site residues.

These analyses collectively revealed that the core structure of all CifB proteins is comprised of four tandem REase fold domains (Fig. 4). Of these, barring type I, in the remaining nine types, the second and fourth REase domains contain the typical active site residues (E…D…EXK…Q), while the first and third domains lack these residues. This suggests that only the second and fourth REase domains are active, while the remaining two are inactive. Analysis of the four-domain architecture of CifB further revealed that the active sites of REase 2 and 4 line a potential substrate-binding groove such that they could cut opposite strands of a DNA duplex (Fig. 4b). However, REase 1 and 3 exhibit a substantial separation from one another and likely contribute to the DNA-binding interface. This might explain why the REase 2 and 4 domains remained active over evolution while REase domains 1 and 3 did not. In the type I CifB proteins (CidB), all four copies lack the characteristic active site residues suggesting that they might be inactive. This is consistent with the proposal that in CidB, the toxicity is mediated by the Ulp1/SMT4 peptidase domain fused to the C-terminus of the four tandem inactive REase domains. Conversely, all the other CifB proteins are predicted to mediate their toxicity (at least in part) via their two active REase domains. A solo version of the 4-REase core is widely seen across different types of CifB proteins (Fig. 4)—importantly, the basal group of CifB proteins (type X) contains only this 4-REase architecture without any other accessory domains (see below). This suggests that the 4-REase architecture represents the ancestral form of the CifB protein, which later accreted further domains.

Curiously, we found that in addition to the type I CidB versions, several other CifB proteins also contain Ulp1/SMT4 and other DUB peptidase and ubiquitin-interacting domains fused to their 4-REase domain core: types VI and VII feature Ulp1/SMT4 just like type I; types V and VIII feature either OTU or Ulp1/SMT4 domains or both; and type IX feature either OTU, PL-OTU, or Ulp1/SMT4 (Fig. 4). The Ulp1/SMT4 domain is usually located at the C-termini, while the OTU and PL-OTU domains are found at the N-termini of CifB proteins. Analysis of their tertiary structures predicted by the AlphaFold2 program (Fig. 4c) revealed no obscuration of the DNA-binding groove of the CifB-REase or the interaction interface between CifB and CifA by these DUB domains. Further, it is notable that several CifB proteins contain DUB peptidase domains despite having catalytically active REase domains. This suggests that these DUB domains might act as coeffectors that facilitate multipronged toxicity (also see below). Alternatively, they might act as “guardian” effectors that protect the CifB protein from counterattack by the host defenses that route it for proteasomal degradation or mislocalization (in the case of K63 modification) by ubiquitination. These proposals are not mutually exclusive because the case of CidB indicates that the optional peptidase domain, which might have originally played a guard function, took over the role of the primary effector in the type I clade. In either case, the multiple associations between CifBs and DUB peptidase domains suggest that they are potentially locked in an arms race with host defenses that target them.

Besides the DUB peptidase domains, we also found that several CifB proteins from types V to IX are often fused to a variable run of ANK repeats. When present at the C-terminus of the protein, these ANK repeats are usually followed by variable domains, such as Latrotoxin-CTD (most common), Tox-SGS, and TcdA/TcdB pore-forming toxin domains (Fig. 4d). The variability of the C-terminal region suggests that these represent a theme comparable to polymorphic toxins and related proteins (Zhang et al. 2012), with ANK repeats as linker/repeat modules coupled to different toxin modules. This raises the possibility that the Latrotoxin-CTD and other ANK-associated C-terminal domains might also function as coeffectors that help augment the toxicity of the core REase domains by targeting other components in the host cell. Additionally, we observed several other instances where the CifB proteins are fused with the typical RHS (Rearrangement HotSpot) repeats and TcdA/B toxin-associated domains that might play a role in their trafficking and deployment in the host cell.

### The Provenance of the CIF-Associated DUB Peptidase Domains

**Fig. 5.**
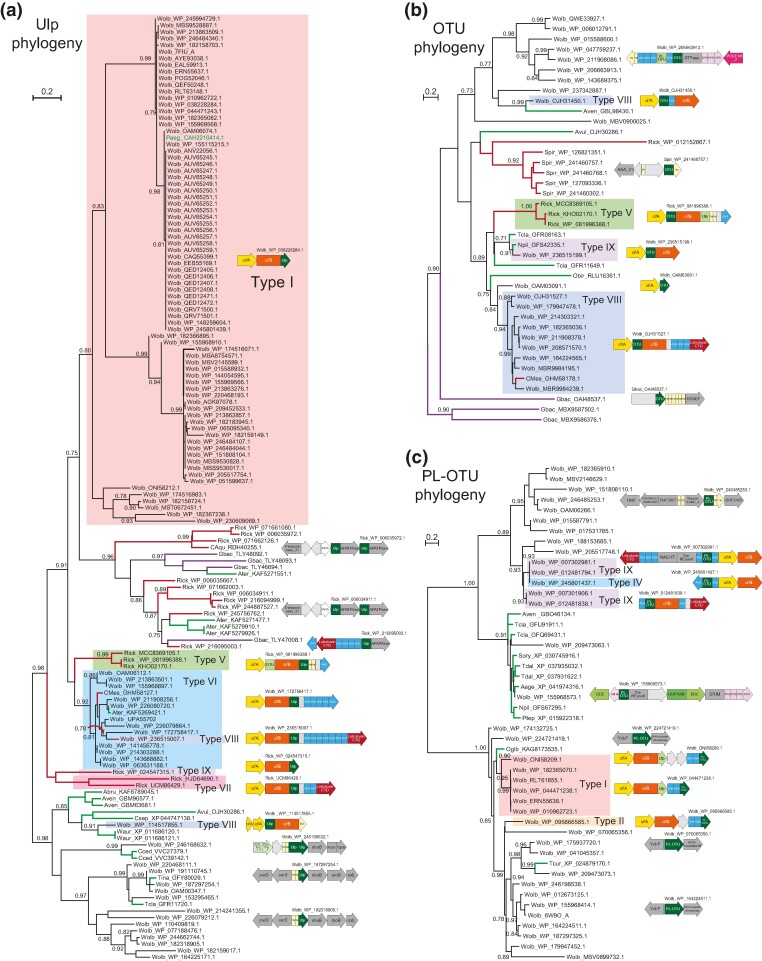
Evolutionary histories of Ulp1/SMT4, OTU, and PL-OTU domains found in CIF loci together with their representative operonic structures and domain annotations. Phylogeny was inferred using the PhyML ML method. The trees with the highest log likelihood were shown with the supporting aLRT SH-like values shown on the major branches. The branches of sequences from *Wolbachia* are shown in black, whereas the sequences from other intracellular bacteria are in red, and the sequences from eukaryotes are in green. Each sequence is represented by the abbreviated species name followed by the NCBI accession number. The operonic structures of selected sequences are shown next to the tree. The major domains of each gene are separately shown as rectangular segments, which are labeled and highlighted in different colors. Species abbreviations used in the figures are as follows: Wolb, *Wolbachia*; Rick, *Rickettsia*; CAqu, “*Candidatus* Aquirickettsiella gammari”; Gbac, *Gammaproteobacteria bacterium*; CMes, “*Candidatus* Mesenet longicola”; Spri, *Spiroplasma*; Page, *Pararge aegeria aegeria*; Ater, *Abscondita terminalis*; Abru, *Argiope bruennichi*; Aven, *Araneus ventricosus*; Avul, *Armadillidium vulgare*; Csep, *Cinara cedri*; Waur, *Wasmannia auropunctata*; Cced, *Cinara ce*dri; Tina, *Trichonephila inaurata madagascariensis*; Tcla, *Trichonephila clavate*; Npil, *Nephila pilipes*; Obir, *Ooceraea biroi*; Sory, *Sitophilus oryzae*; Tdal, *Teleopsis dalmanni*; Aage, *Aricia agestis*; Ptep, *Parasteatoda tepidariorum*; Ogib, *Oedothorax gibbosus*; Tcur, *Temnothorax curvispinosus*.

Given that the Ulp1/SMT4, OTU, and PL-OTU domains are specifically involved in the processing and deconjugation of ubiquitin/Ubl in eukaryotic cells, we were interested in understanding the process of their incorporation into the CifB proteins or into the CIF loci. Accordingly, we performed comprehensive phylogenetic analyses of the CIF-associated versions of these proteins, together with their homologs found in eukaryotes and bacteria. The tree topology of the Ulp1/SMT4 domains (Fig. 5a) showed that versions from the CifB proteins are not monophyletic. Specifically, the Ulp1/SMT4 domains from the type I CifB proteins are related to the versions from other intracellular symbiotic/parasitic bacteria, including *Rickettsia* and versions from the genomes of some insect species (e.g. the beetle *Abscondita terminalis*). Ulp1/SMT4 domains from types V and VI form one monophyletic clade, while those from types VII and IX CIF loci form another monophyletic clade. Interestingly, Ulp1/SMT4 domains from type VIII loci were either nested within the radiation of type VI homologs or formed a distinct clade with sequences from several insects, such as the little fire ant *Wasmannia auropunctata*. Further, these type VIII sequences also group with a large assemblage of Ulp1/SMT4 domains from non-CIF loci encoded elsewhere in *Wolbachia* genomes.

The tree of the OTU domain (Fig. 5b) shows a similar pattern. Majority of the type VIII OTU domains form a monophyletic clade with cognates from types V and IX CIF loci. However, one type VIII OTU domain instead forms a clade with a cognate domain from the orb spider *Araneus ventricosus* (GBL98430.1). These are further nested within the radiation of OTU domains from *Wolbachia* non-*cif* loci that are predicted to encode other host-manipulating effectors (i.e. WP_206663913.1). In the case of the PL-OTU domains (Fig. 5c), the exemplars from types IV and IX CifB proteins form a monophyletic clade, whereas those associated with types I and II loci form distinct clades. All of these are nested within a large radiation of PL-OTU domains from non-CIF *Wolbachia* loci that are predicted to encode other host-manipulating toxins. Notably, the branch, including the types IV and IX CifB proteins, also includes genomic PL-OTU homologs from a wide range of spiders, moths, and beetles.

These observations allow us to draw several key inferences regarding the provenance of the DUB peptidase domains in the CifB proteins or associated with the CIF loci: (i) despite similar contexts, the paraphyly of the DUB domains in the CIF loci indicates that they have undergone repeated displacement and/or accretion to reconstitute functionally equivalent domain architectures and gene neighborhoods. These points to a major role for a recombinogenic process in their evolution. (ii) The DUB domains from the CIF loci are related to cognate domains from non-CIF loci predicted to encode other host-manipulating effectors present across the *Wolbachia* genomes. This indicates that comparable biochemical processes, such as interference with the host Ub/Ubl system, might be used both in CI and other host-targeting activities of bacterial endosymbionts. Thus, effectors used in other *Wolbachia* conflict systems might be drafted into the CI system. (iii) Not only are the DUB domains drawn from other *Wolbachia*-encoded conflict systems, but they are also derived from similar loci in other endosymbiotic/parasitic bacteria and host genomes. Thus, the recombinogenic process involved in generating a variable repertoire of DUB domains at the CIF loci not only draws from other *Wolbachia* genomic loci but also from a larger pool of the versions of these DUB domains acquired through a web of lateral transfer with other bacteria and their hosts.

### Evidence for the Continued and Ongoing Recombination between the Core REase and Ulp1/SMT4 Domains in Type I CifB Proteins

**Fig. 6.**
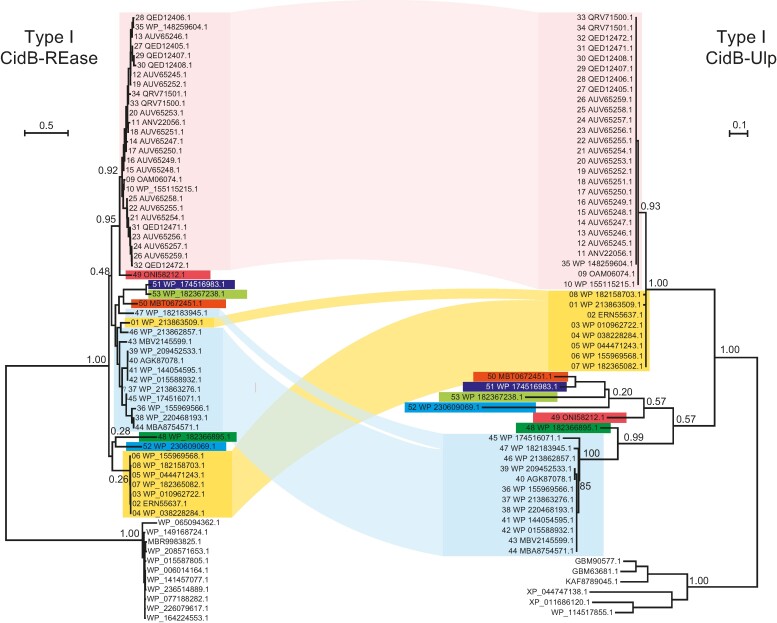
Inconsistent evolutionary histories of the coupled core REase and Ulp1/SMT4 domains of type I CifB proteins. The phylogeny was inferred using the PhyML ML method. The same background color indicates the correspondence of the REase and Ulp1/SMT4 domains from the same proteins.

The above phylogenetic analyses showed that diversification between and within different types of CIF loci involved recombination events that brought together disparate DUB domains with the 4-REase core. We next investigated if similar recombination events might be taking place even within a clade when the same domain architecture is maintained by a CifB protein. For this purpose, we chose the widely represented type I CifB protein and constructed separate phylogenetic trees for the REase and coupled Ulp1/SMT4 domains. Remarkably, a comparison of the respective tree topologies indicated they were not congruent—the higher-order grouping of different type I Ulp1/SMT4 clades was notably different from their cognate REase domains (Fig. 6). This implied that different type I loci have undergone recombination with each other resulting in mosaicism in their REase and Ulp1/SMT4, with each showing distinct ancestries. This observation suggests that the CifB genes are potential hotspots for recombination, and the process shapes their diversity probably in face of the arms race with host defenses. These observations are consistent with a previous study (Bonneau et al. 2018a) in which both *CifB* and *CifA* in several wPip strains were found to undergo extensive recombination.

### Evidence for Pervasive Recombination across Multiple Frequently Occurring Genes in the CIF Neighborhoods

**Fig. 7.**
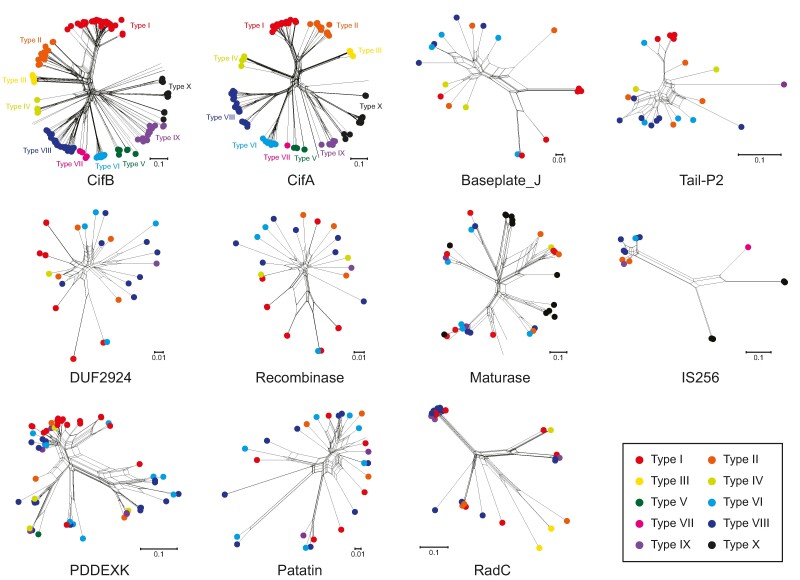
Phylogenetic networks of 11 gene families found on the CIF loci. The network trees are generated using the SplitsTree4 program with default parameters. Corresponding to Fig. 2, the sequences are highlighted according to the type of their CIF loci. In the phylogenetic network tree, each tip indicates a single protein sequence, each edge represents the evolutionary relationship of nodes, and each internal node represents the ancestral node. The two sets of parallel edges (in the form of a box) indicate alternative inconsistent evolutionary trajectories among the parts of the gene as caused by recombination (in this study).

Given the above observations, we proceeded to explore if a similar recombinogenic process might not just operate on CifA/B but across the other genes associated with this neighborhood. For this purpose, we used phylogenetic network analysis, a powerful technique that allows the identification of inconsistent evolutionary histories among the different parts of the same gene or different genes from the same locus (Atyame et al. 2011). Accordingly, we chose 11 distinct protein families that are associated with different CIF loci and performed a SplitsTree analysis (Huson and Bryant 2006) on them (Fig. 7). As a control, we used both CifB and CifA—keeping with our expectations, we recovered complex tree networks indicative of recombination shaping their evolutionary trajectories. However, despite this, there was congruence in the higher-order topologies of CifA and CifB (Fig. 7), which in turn was equivalent to the topologies we had earlier obtained in ML/NJ trees (Fig. 2). This indicated that despite the recombination, CifB and CifA continue to coevolve as a single unit.

For the remaining nine protein families associated with the CIF loci, we included different functional categories: (i) predicted effectors such as RadC, Patatin; (ii) transposons such as Recombinase carried by prophages, IS256 transposase, Maturase (a RNA-binding enzyme required for processing of group II intron-like retroelements), the transposase REase domain (Pfam: PDDEXK_2), as well as the RAMA DNA-binding domain; and (iii) structural components of prophages: Baseplate J and Tail P2 proteins. The trees of these families reveal: (i) sequences from disparate CIF loci types grouping together and (ii) many parallel edges (boxes of edges). These features indicate that various genes in the different CIF genomic neighborhoods might have been exchanged potentially through lateral transfer between different *Wolbachia* strains or between the multiple copies present in the same genome. Further, the phylogenetic network structure indicates that these genes are potentially undergoing recombinogenic gene conversion by segments derived from more distant homologs (Zhang et al. 2016; Bonneau et al. 2018a). Thus, not just the CifA/B genes but also the broader CIF neighborhoods are recombination hot spots in the *Wolbachia* genomes.

## Discussion

### The Evolutionary Processes for the Diversification of the CIF Loci

Our comprehensive investigation of the CIF loci, which are key molecular determinants of *Wolbachia* CI, has helped uncover their genomic diversity and potential evolutionary mechanisms. We extend the previously known CIF loci and show that they belong to ten distinct types. These gene neighborhoods, including the CIF genes themselves, display a striking variation in both copy number and gene composition/organization. We present the evidence for extensive recombination and domain architectural variation in CifB. A similar recombinogenic variation extends to several other genes in the larger genomic neighborhoods defining the CIF loci. A case-in-point is the remarkable variability in terms of the accreted or displaced DUB peptidase domains belonging to different families that are coupled to the 4-REase core of CifB. We propose the “guardian” hypothesis for these DUB domains, wherein they originated as deubiquitinating enzymes that protect the core effector from proteasomal degradation or mislocalization by Ub modification. In the case of the type I CifB, this guardian peptidase domain of the Ulp1/SMT4 family took over as the primary effector domain, with the concomitant inactivation of all the REase domains.

*Wolbachia* has been proposed to be one of the intracellular bacterial genomes that show a high degree of recombination (Klasson et al. 2009; Atyame et al. 2011; Gillespie et al. 2018; Scholz et al. 2020). However, a systematic screening of the 210 conserved orthologous groups among 33 *Wolbachia* strains only revealed a few instances of intergenome recombination (Wang et al. 2020). This suggests that the recombination across the genomes is uneven. While the *Wolbachia* CIF loci have previously been shown to be borne by prophages, we find that this situation is not universal. However, we find a pervasive association of the CIF genomic neighborhoods with a diverse array of mobile elements, both DNA transposons and retrotransposons. Hence, we propose these are the primary mediators of both recombination at the CIF loci and the extensive lateral transfer that characterizes them. We present evidence for the maintenance of this recombination across evolutionary timescales suggesting that it is a major driver of the diversification of these loci under an arms race.

### Functional and Evolutionary Implications for CifB and CifA Interactions

This study also sheds light on the mode of interaction between CifB and CifA. Several models are currently available to explain the mechanism of CI, with the primary ones being TA, host-modification (H-M), and two-by-one models. In the TA model, CifB is proposed to be associated with the highly condensed sperm genome and to enter the egg during fertilization. Shortly after the protamine is shed from the sperm DNA, the toxic effects of CifB start manifesting, resulting in the defective mitotic condensation of male chromosomes. This causes a subsequent chromosome segregation failure during the zygotic mitosis resulting in mortality. However, if the female is also infected by the same *Wolbachia* strain, then CifA is also present and acts as an antidote that counters the toxic effect of CifB (Bonneau et al. 2018a; Beckmann et al. 2019). Alternative models like the two-by-one model suggest that both CifB and CifA, introduced alongside the sperm pronucleus, play a role in causing the mortality associated with CI. However, in a female infected by the same strain, the egg-borne CifA alone is necessary and sufficient to rescue CI (LePage et al. 2017; Shropshire et al. 2018; Shropshire and Bordenstein 2019).

Our observations indicate that in addition to the strictly preserved operonic association between CifB and CifA, they also share congruent evolutionary histories, despite extensive recombination. Even though the component domains of CifB show considerable evidence for shuffling and exchange between the different types of CIF loci, the corresponding CifAs show a coevolutionary connection. This is a strong indication of them functioning as a unit where the function of one is “tracked” by the other in the course of evolution. Studies on numerous effectors across different biological conflict systems show that they fall into relatively few functional “guilds” (Aravind et al. 2022). One of these are endonuclease effectors that cleave DNA or RNA—indeed REase domains are seen as effectors across diverse conflict systems such as polymorphic toxins (Zhang et al. 2011, 2012), other interorganismal conflict effectors (e.g. CR-effectors; Zhang et al. 2016), restriction–modification (Xu et al. 2000) and other systems involved in antiviral/plasmid defense (Iyer et al. 2013; Burroughs et al. 2015). Thus, the presence of two active REase domains in the majority of the CifB components indicates that these are indeed DNA-targeting toxins that evince their toxicity by cleaving the chromosome and triggering segregation defects. Moreover, the CifB components also show polymorphism via the recruitment of other toxin domains. In contrast, CifA is nonenzymatic—indeed, comparable superstructure-forming repeats have been observed as immunity proteins or negative regulators in other effector systems, such as polymorphic toxin systems (Zhang et al. 2012).

The two-gene genomic organization of the CifA/B system is again reminiscent of various effector systems such as polymorphic toxins and toxin–antitoxin systems, wherein predominantly noncatalytic immunity proteins or antitoxins act as antidotes for the enzymatic effectors coded by these systems. Hence, viewing the CifA/B systems as a comparable TA dyad would be natural. However, if the two CIF genes function cooperatively as proposed by the host-modification or two-by-one models, we would expect fusions of the CifA HEAT repeats with the core 4-REase effector module—this is observed for other toxin domains with potential coeffector or guardian effector functions. Strikingly, we did not recover even a single instance of such a fusion. Thus, taken together, all the above lines of evidence tilt the balance in favor of the TA model for the CifA/B interaction in CI.

### The Ultimate Origin of the CifB Endonucleases

Our sequence and structural analysis (supplementary figs. S3 and S4, Supplementary Material online) revealed that the REase domains of CifB might be related to the CR-REase7 clade found in CR-effectors of eukaryotes and related bacterial proteins of transposon origin (Zhang et al. 2016). The iterative PSI-BLAST searches retrieved the CR-REase7 homologs as the closest homologs outside CifB members. Both share a synapomorphic histidine (H), located three residues downstream of the conserved REase active site glutamate (E), and another glutamate (E) on the first β-strand of the core, which together contribute to the active site. Additionally, close relatives of CifB REases are present in mobile CR-effector-like elements that combine the REase domain with an N-terminal CR-ATPase8 domain (Zhang et al. 2016). This architecture, combining CR-NTPase8 and CR-REase7, is also observed in mobile elements exemplified by the beetle *Tribolium castaneum* Medea1, related to CR-effectors and similar bacterial proteins (Lorenzen et al. 2008; Zhang et al. 2016). Remarkably, the Medea1 element causes postzygotic embryo killing, known as Maternal-Effect Dominant Embryonic Arrest (MEDEA), which also resembles CI. Considering that CR-effectors and related elements have a broader distribution than CIF systems, our observations support the hypothesis that the REases of the CIF system evolved from a CR-effector-like mobile element, potentially mediating embryo killing similar to the Medea1 systems (Beckmann et al. 2017).

### Novel Predicted Effectors in *Wolbachia*

Our analysis helped identify several other components in the CIF genomic neighborhoods, as well as homologs elsewhere in the *Wolbachia* genomes that have the potential to function as host-manipulating effectors. These chiefly include many DUBs of different folds, potential neurotoxins (e.g. latrotoxin), the RadC-type JAB domain of the deaminase-like fold, DNA glycosylase/endonuclease V, as well as the Patatin-like phospholipase family protein. This is in keeping with the previously reported clustering of disparate conflict systems in various bacterial genomes in the form of variable mobile defense islands (Zhang et al. 2011; Zhang et al. 2012; Gillespie et al. 2018). In addition to CI, *Wolbachia* manipulates its host's reproductive biology through selective male killing, feminization, and parthenogenesis (Werren et al. 2008). The molecular determinants underlying these influences on host biology are largely unknown. Furthermore, not all aspects of CI are accounted for by the CifB/A operon (Baiao et al. 2021). Therefore, the novel effectors uncovered in this study might provide the framework for a directed approach to investigate the lesser-understood aspects of the interactions of *Wolbachia* and other intracellular bacteria and their hosts.

## Supplementary Material

evae171_Supplementary_Data

## Data Availability

All genome and protein sequences analyzed in this study were downloaded from the NCBI GenBank database. The accession numbers of the sequences can be found in supplementary data, Supplementary Material online.
