# Supplementary material for: Genomic Underpinnings of Cytoplasmic Incompatibility: CIF Gene-Neighborhood Diversification Through Extensive Lateral Transfers and Recombination in Wolbachia
Source: Genome Biol Evol. 2024 Aug 6;16(8):evae171. doi: 10.1093/gbe/evae171 (PMC11342252; doi:10.1093/gbe/evae171)

Supplementary Materials for

**Genomic underpinnings of Cytoplasmic Incompatibility: CIF gene neighborhood diversification through extensive lateral transfers and recombination in *Wolbachia***

Yongjun Tan^a^, L. Aravind^b^, Dapeng Zhang^a,c^

^a^Department of Biology, College of Arts and Sciences, Saint Louis University, St. Louis, MO 63103, USA

^b^National Center for Biotechnology Information, National Library of Medicine, National Institutes of Health, Bethesda, MD 20894, USA

^c^Program of Bioinformatics and Computational Biology, College of Arts and Sciences, Saint Louis University, St. Louis, MO 63103, USA

Corresponding author: Dapeng Zhang

E-mail: dapeng.zhang@slu.edu

Content:

1. Supplementary Figure 1. Phylogenetic analysis of the CifB using PhyML, Neighbor-Joining, Bayesian Inference, and FastTree methods.
2. Supplementary Figure 2. Phylogenetic analysis of the CifA using PhyML, Neighbor-Joining, Bayesian Inference, and FastTree methods.
3. Supplementary Figure 3. Multiple sequence alignment (MSA) of the CifB REase2 domain, CifB REase 4 domain and CR-REase-7 domain.
4. Supplementary Figure 4. Structural analysis of CifB REase2 domain and CR-REase-7 domain.

**Supplementary Figure 1. Phylogenetic analysis of the CifB using PhyML, Neighbor-Joining, Bayesian Inference, and FastTree methods.**


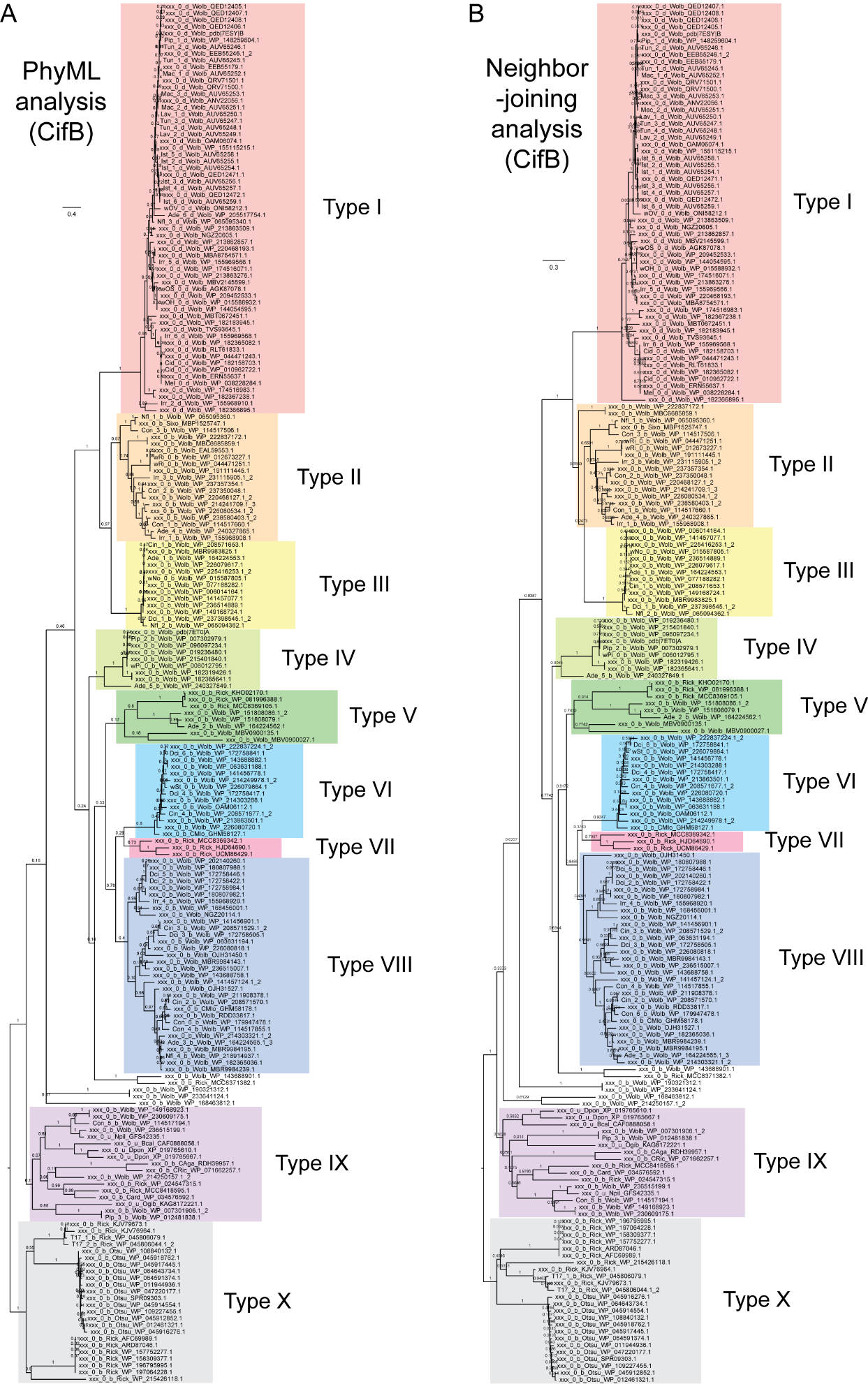


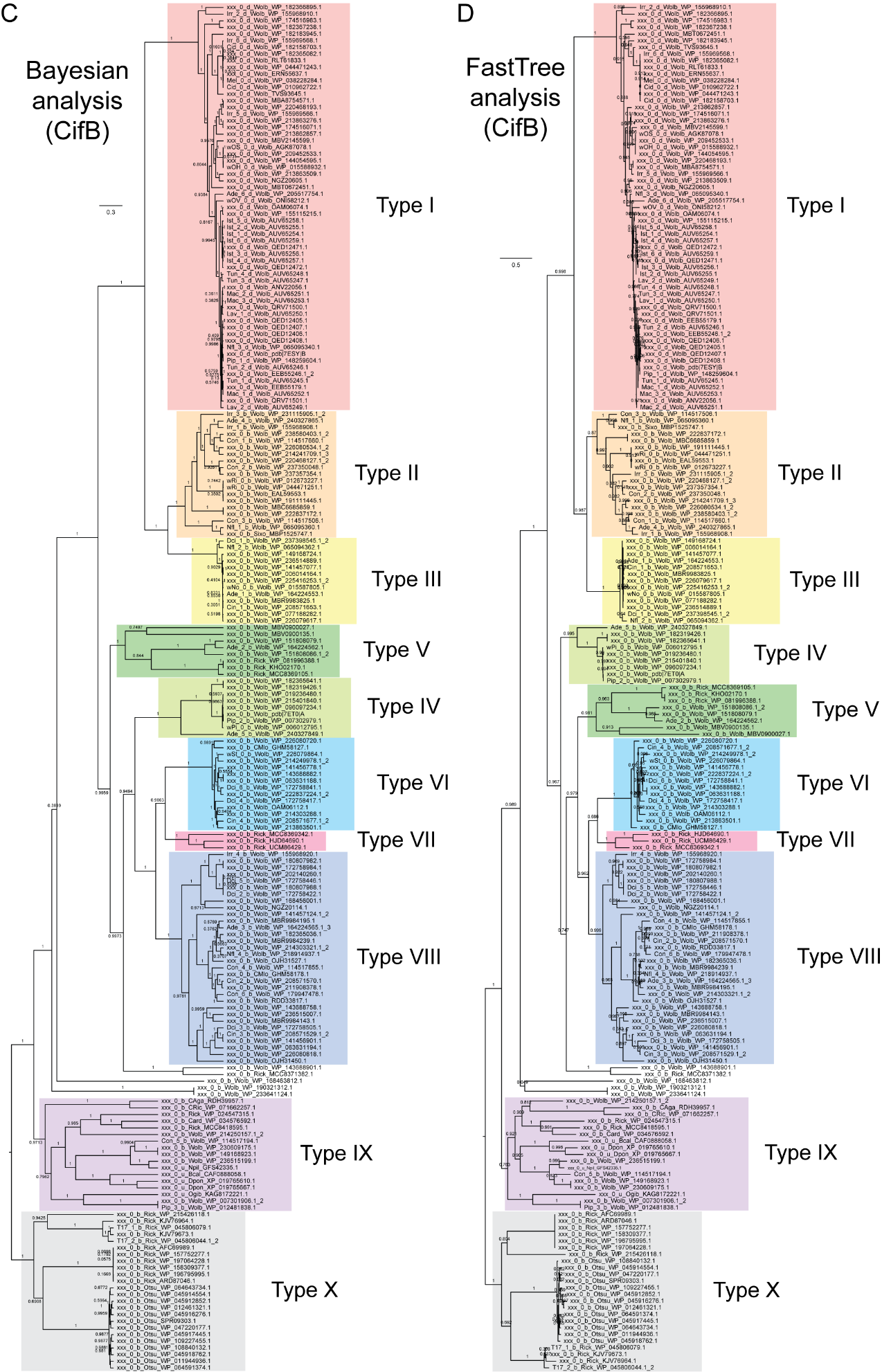
 **Supplementary Figure 2. Phylogenetic analysis of the CifB using PhyML, Neighbor-Joining, Bayesian Inference, and FastTree methods.**


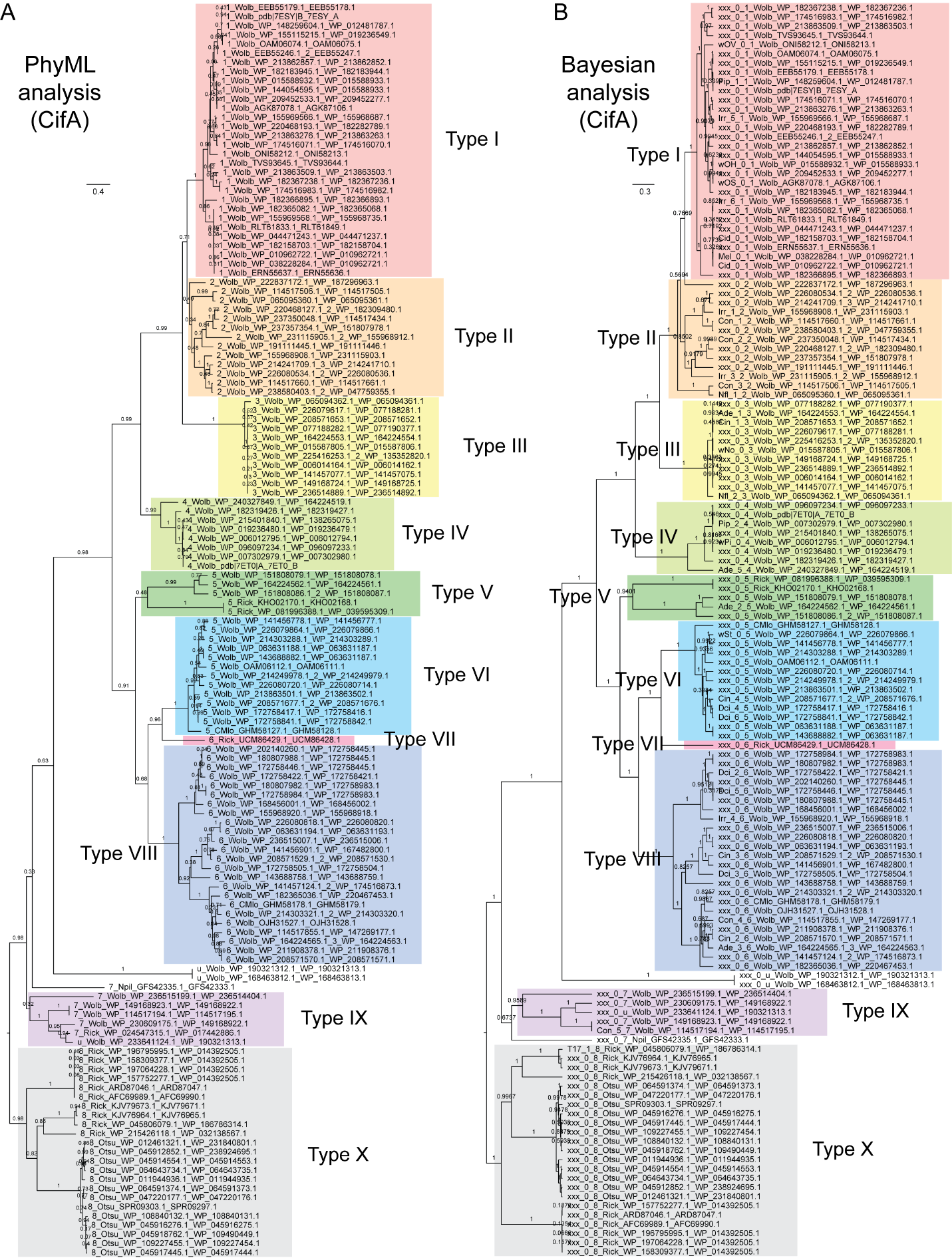


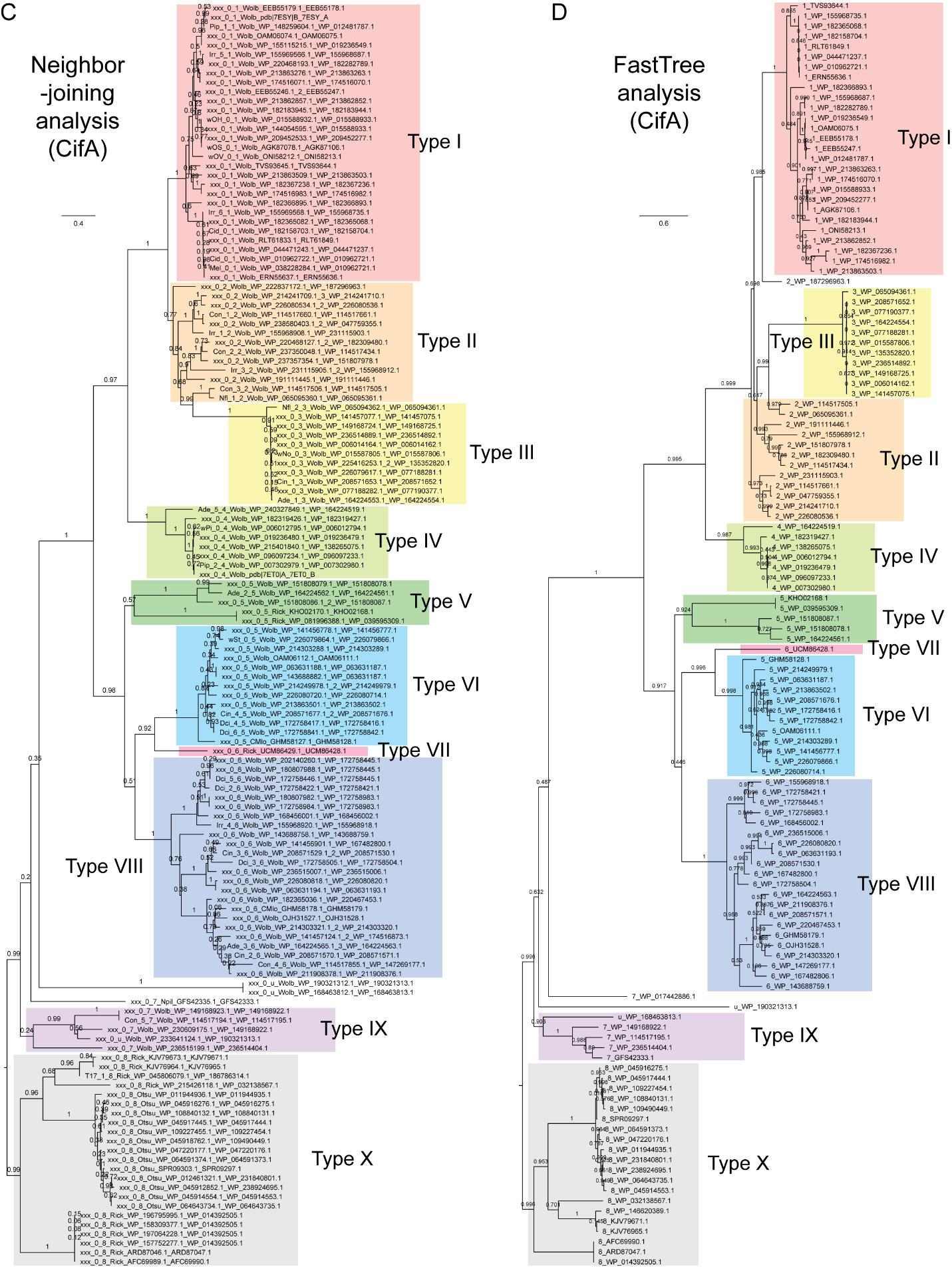


**Supplementary Figure 3. Multiple sequence alignment (MSA) of the CifB REase2 domain, CifB REase 4 domain and CR-REase-7 domain.** The CifB sequences in MSA are labelled by their types (2, …, 9, X) and the REase domain (2 or 4) followed by their species abbreviation and NCBI accession number, while CR-REase-7 REase sequences are labelled by their domain name abbreviation (CR7) followed by their species abbreviation and NCBI accession number. The secondary structure is shown above the MSA, while the consensus result calculated by a custom perl script is displayed below the MSA. The MSA was colored according to the consensus in 80% of the sequences based on 7 classes of residues. Coloring theme: the typical catalytic residues of the REase are highlighted in red background with white font color; the two new family-specific residues are highlighted in hotpink background with white font color; any residues present above the threshold are highlighted in black background with white font color and bold font style; hydrophobic (h) in yellow background; aromatic groups (a) in orange background; big amino acids (b) in light grey background; small amino acids (s) in green background; polar (p) group in blue background.

Secondary Structure **HHHHHHHHHHHHHHHH** **EEEEEEE** **EEEEEE** **EEEEEE** **HHHHHHHHHHHHHHHHH** **EEEE EEEE**

X_2_Rick_WP_157752277.1 SSEASYHGFVYGLFVLNYRDKY----WIDTSVEQNSG-----RGVLDLSLLFKKNS--DGVRAEYAPHIILEFKQ-------GEQLCQSAQTAIRQIRHKGYAY--KL-IGRTKSKYAIG-VGINFNREDP

X_2_Rick_WP_158309377.1 SSEASYHGFVYGLFVLNYRDKY----WIDTSVEQNSG-----RGVLDLSLLFKKNS--DGVRAEYAPHIILEFKQ-------GEQLCQSAQTAIRQIRHKGYAY--KL-IGRTKSKYAIG-VGINFNREDP

X_2_Rick_WP_196795995.1 SSEASYHGFVYGLFVLNYRDKY----WIDTSVEQNSG-----RGVLDLSLLFKKNS--DGVRAEYAPHIILEFKQ-------GEQLCQSAQTAIRQIRHKGYAY--KL-IGRTKSKYAIG-VGINFNREDP

X_2_Rick_WP_197064228.1 SSEASYHGFVYGLFVLNYRDKY----WIDTSVEQNSG-----RGVLDLSLLFKKNS--DGVRAEYAPHIILEFKQ-------GEQLCQSAQTAIRQIRHKGYAY--KL-IGRTKSKYAIG-VGINFNREDP

X_2_Otsu_WP_011944936.1 TSESRYHGFVYGALVLNFKQKY----SMDVYVEQSAG-----RGYLDLYLLCKHYNHNQIA-NADAVKIIAEFKN-------GNNKYPSVADAIKQVEDRCYA---QL-VGRTQSNKTII-VGADFGDITK

X_2_Otsu_WP_064591374.1 TSESRYHGFVYGALVLNFRQKY----SMDVYVEQSAG-----RGYLDLYLLCKHYNH(4)NADANAVKIIAEFKN-------GNNKYPSVADAIKQVEDRCYA---QL-VGRTQSNKTII-VGADFGDITK

X_2_Otsu_WP_012461321.1 TSESRYHGFVYGALVLNFKQKY----SIDVYVEQSAG-----RGYLDLYLLCKHYNQ(4)NADANAVKIIAEFKN-------GNNKYPSVSDAIKQVEDRCYT---QL-IGRTQSNKTII-VGADFGDITK

X_2_Otsu_WP_045912852.1 TSESRYHGFVYGALVLNFRQKY----SMDVYVEQSAG-----RGYLDLYLLCKHYNHNQIA-NADAIKIISEFKN-------GNNKYPSVADAIKQVEDRCYA---QL-IGRTQSNKTII-VGADFGDITK

9_2_CAga_RDH39957.1 RKEAFSHGFLYGSLSLNFKNQY----RLDAYVERVAG-----NGYADLVMISRVN--------ANAIPILIELKA----------GTATVRDAVAQIKKIGYFQ--HEPSLRTYHPNGVI-AGVNFNSGSI

9_2_CRic_WP_071662257.1 STEAFSHGFLYGSLSLNFKNRY----HIDCYVERISG-----NGYTDIMMTSRANNR-----RSNSIAIIIELKA----------GIATAREAIDQIQNKGYFQ--HVLSLRTYSETVII-AGVNFNLADD

9_2_Rick_WP_024547315.1 KSEAAYHGFSYGFFVLNYKYKY----ALDCYVERRSG-----KGYMDLILLSRID-------NNSPIPIIVELKA----------EQFSADSAIEQIHNHGYIY--KLPSVRTSARNAVI-VGVNYNQKLL

9_2_Rick_MCC8418595.1 TSESAYHGSIYGFFRLNYKYKY----ALDCYVERIAG-----KGYADIMLLSRKISD(15)DKKQFIPVIVEVKA----------R-TGADIAIEQINKSGYIY--NLPNIRTNAKEAVI-VGIDFNTKAK

9_2_Dpon_XP_019765667.1 KSEAAFQGFLYGILSLNFKYHY----YLDIYVERIAG-----KGYADLILLSRKDN--SHNKNWRAIPIVIEFKA----------DETSSASAIDQAKGTGYLY--NL-SMRTISDQAVI-VGINSKIDSD

9_2_Bcal_CAF0888058.1 NSESGFHGFFYGALSLNFKENF----NLDISVERIAG-----KGYADLIVYSRKGH--DGNLNLNAVPIIVEFKS----------DRTSPSDAAYQATGTGYLY--NL-SVRTLSKIALV-IGINSKLPIE

9_2_Wolb_WP_236515199.1 NSEAAYHGFVYGALVLNFKYRY----GLNCYVERAAG-----NGRADLILMSRTKD-ANGRINPKPIPVVVEFKA----------KNHTASEAIKQIKDKGYLY--NL-SVRTRAKGAVI-VGVNPAQSDA

9_2_Npil_GFS42335.1 NSEAAYHGFFYGALALNFKYRY----GLNCYVERTAG-----NGRADLILMSRTKD-ANGRINPKPIPVVVEFKG----------EGYTADDAIAQIKNKGYLY--NL-SVRTKAKDVVI-AGVSLTGEPQ

9_2_Wolb_WP_114517194.1 NSEAAYHGFHYGALALNFKYRY----DLNCYVERTAG-----NGRADLIFISRTKD-ANGRMNSKPTPVVVEFKS----------GDHAVDEAIAQIENKGYLY--NL-NVRTKAEEAVI-VGVSRAKVKT

9_2_Wolb_WP_149168923.1 NSEAAYHRFVYGALALNFKYRY----GLNCYVERAAG-----NGRADLILMSRTKD-ANGRINPKPVPVVVEFKG----------EGHTADEAITQIKNKGYLY--NL-SIRTKAEEAVI-VGISYAEVKT

9_2_Wolb_WP_230609175.1 NSEAAYHGFVYGALALNFKYRY----GLNCYVERAAG-----NGRADLILMSRTKD-ANGRINPKPVPVVVEFKG----------EGHTADEAITQIKNKGYLY--NL-SIRTKAEEAVI-VGISYAEVKT

8_2_Wolb_WP_172758984.1 VREAAHHGFIAGALV-NFRYRY----NLRVYLEQFAG-----RGYADIVLVPRGKD-----RSLNAVPIIIELKAGT-------SSGTTPNNALEQAKDYAKGFQPNTMRVLTISDNLLC-VGLNLDSTES

8_2_Wolb_WP_180807982.1 VREAAHHGFIAGALV-NFRYRY----NLRVYLEQFAG-----RGYADIVLVPRGKD-----RSLNAVPIIIELKAGT-------SSGTTPNNALEQAKDYAKGFQPNTMRVLTISDNLLC-VGLNLDSTES

8_2_Wolb_WP_155968920.1 AREAAHHGFLAGALV-NFRYRH----NLRVYLEQFAG-----RGYADIVLVPRGKG-----RSLNAVPIIIEMRAATK(7)IKESSETTPAAALKQAKDYAKGFQPNTMRVLTVSDNVLC-VGLNLDSTES

8_2_Wolb_WP_168456001.1 VREAAHHGFIAGALV-NFRYRH----NLRVYLEQFAG-----RGYADIVLVPRGKN-----RLPNAVPIIIELKAGI-------GHGKELDDALEQAKGYAKGFQPNTTRVLTISDNVLC-VGLNLDSTKK

8_2_Wolb_WP_226080818.1 LRETAHHGFIAGALV-NFRYRH----NLRVYLEQFAG-----RGYADIVLVPRGKD-----RSLNAIPIIIEMKAATK(7)IGEKSGTAPTTALKQAEDYTKGFQPNVMRILTTANDMLC-VGVNLDHPSP

8_2_Wolb_WP_236515007.1 TREAAHHGFIAGALV-NFRYKH----NLRVYLEQFAG-----RGYADIVLVPRGKD-----RALNAIPVIIEMKAATK(7)IGEKSGTTPAAALKQAEDYTKGFQPNVMRVLTTANDILC-VGVNLDHPSP

8_2_Wolb_WP_143688758.1 AREAAHHGFMAGALV-NFRYRH----NLRVYLEQLAG-----RGYADIILVPRGKD-----RSLNAIPIIIELKAATK(12)KDGSQTTPAVALKQAEDYTKGFQPNVMRVLTTANDILC-VGVNLDYPSA

8_2_Wolb_WP_211908378.1 AREAAHHGFAVGVFM-NFHYRY----NFRVYPEQFAG-----RGYADIILLARGPD-----RALDSIPIIIELKAGA-------GSNATPDKALQQAEKYAQGFQPNIQRVLTTADDILC-VGVNLDNPSP

8_2_Wolb_WP_208571570.1 AREAAHHGFMVGVFM-NFHYRY----NLRVYPEQFAG-----RGYADIILLARGPD-----RALDSIPIIIELKAGA-------GSNATPDKALQQAEKYAQGFQPNVQRVLTTADDILC-VGVNLDNPSP

8_2_CMlo_GHM58178.1 AREAAHHGFMVGVFM-NFHYRY----NLRVYPEQFAG-----RGYADIILLARGTN-----RALNSIPIIIELKAGA-------GPYATPGKALQQAEKYAKGFQPNVMRVLTTADEILC-VGVNLDSSSP

8_2_Wolb_WP_179947478.1 AREAAHHGFMVGVFM-NFHYRY----NLRVYPEQFAG-----RGYADIVLVPRGPD-----RALNSIPIIIELKAGA-------DSNSTPDKALEQAEKYAQGFQPNVQRVLTTADNILC-VGVNLDHPSP

8_2_Wolb_WP_114517855.1 AREAAHHGFIAGALN-NFRYRY----NLRAYLEQFAG-----RGYADIVLLPRGPD-----RALNSIPIIIELKAGN-------GPNLTPDKALEQAEKYAQGFQPNVQRVLTTANNILC-VGVNLDHPSP

7_2_Rick_HJD64690.1 AREADEHGFVAGIFN-NFKYRE----NVHGYLEQFAG-----TGYADMVLLVRGSD-----RTTKSIPIIMEFKAGT-------GAGTNADDALKQVEEYTKGFQPNNMRILSLADNILC-VGMNLDSPSD

7_2_Rick_UCM86429.1 AKEANEHGFVAGIFN-NFRYRE----NAHVYLEQFAS-----RGYADIILLVRGAD-----RSTNSIPIIIELKAGS-------GISTTPSSALEQATKYSQGFQPNNKRILSFSDNILC-IGMNLDNLAN

6_2_Wolb_WP_141456778.1 AREAAQHGFVAGIFD-NFRYRD----NTKLYLEQFAS-----GGYADIVLLVRGPN-----RAIDSVPILIELKAGT-------EGQVDPSDALRQAEDYIKGFRPNKMRILTNADNAIA-VGLNLDNAEP

6_2_Wolb_WP_226079864.1 AREADQHGFVAGIFD-NFRYRD----NTKLYLEQFAG-----GGYADIVLLVRGPN-----RAVDSVPILIELKAGT-------EGQVDPSDALRQAEDYIKGFRPNKMRILTNAGDAIA-VGLNLDNAEP

6_2_Wolb_WP_172758417.1 AREADQHGFVAGVFD-NFRYRD----NTKLYLEQFAG-----GGYADIVLLVRGPN-----RAVDSVPILIELKAGT-------EGQVDPSDALRQAEDYIEGFRPNKMRILTNADNAIA-VGLNLDNAEP

6_2_Wolb_WP_214303288.1 AREADQHGFVAGIFD-NFRYRD----NTKLYLEQFAG-----GGYADIVLLVRGPN-----RAVDSVPILIELKAGT-------EGQIDPSDALEQAKDYIKGFRPNKMRILTNADNAIA-VGLNLDDAEP

6_2_Wolb_WP_213863501.1 AREAGQHGFVAGMFD-NFRYRD----NTKLYLEQFAG-----GGYADIVLLVRGPN-----RAVDSVPILIELKAGT-------VGEVTPNDALKQAEGYIEGFRPNKMRVLTNADNAIA-VGLNLDYTES

6_2_Wolb_WP_226080720.1 AREAGQHGFVAGIFD-NFRYRD----NTKIYLEQFAG-----GGYADIVLLVRGPN-----RAIDSVPILIELKAGT-------EGQVNPSDALRQAEDYIEGFRPNKMRILTNADDAIA-VGLNLDSTEP

6_2_CMlo_GHM58127.1 AREADQHGFVVGVFD-NFRYRD----NTKIYLEQFAG-----GGYADIVLLVRGSN-----RAIDSVPILIELKAGT-------KGQVTPSDALKQAKDYVKGFRPNKMRVLTNTGDVIA-VGLNLDFKKP

5_2_Rick_KHO02170.1 ALEAADHGFTAGALI-NFKYRY----NLELYLELLLGI----EGYIDIALLVRGSQ-----RVKKAMPILAEIKTGQ-----EQGGRTNPQQALRESESYTQGLQQNNRPFVTLADRVIR-VGFNMDYTNP

5_2_Rick_WP_081996388.1 ALEAADHGFTAGALI-NFKYRY----NLELYLELLLGI----EGYIDIALLVRGSQ-----RVKKAMPILAEIKTGQ-----EQGGRTNPQQALRESESYTQGLQQNNRPFVTLADRVIR-VGFNMDYTNP

5_2_Rick_MCC8369105.1 ALEAADHGFTAGALI-NFKYRY----NLELYLELLLGI----EGYIDIALLVRGSE-----NVKKAMPILVEIKTGQ-----EQNGRTNPQQALREAESYTQGLQQNNRPFVTLADRVVR-VGFNMDYANP

4_2_Wolb_pdb|7ET0|A GKESSHHGFLAGFLI-NFKYRF----HLKLYLELFAG-----KGYADIILLVRGSD-----KSLSSIPIIIELKAGT-------GEISTVIKALKQAQDYVKGSFSNSIRMITIANEAIC-VGLNFDMVHH

4_2_Wolb_WP_006012795.1 GKESSHHGFLAGFLI-NFKYRF----HLKLYLELFAG-----KGYADIILLVRGSD-----KSLSSIPIIIELKAGT-------GEISTVIKALKQAQDYVKGSFSNSIRMITIANEAIC-VGLNFDMVHH

4_2_Wolb_WP_182319426.1 SKESSHHGFLAGFLI-NFKYRF----HLKLYLELFAG-----KGYADIILLVRGSD-----KSLSSIPIIIELKAGT-------GEISTVIKAFKQVQDYVKGYFSNSIRMITIANKAIC-VGLNFEMVHH

4_2_Wolb_WP_182365641.1 SKESSHHGFLAGFLI-NFKYRF----HLKLYLELFAG-----KGYADIILLVRGSD-----KSLSSIPIIIELKAGT-------GEISTVIKAFKQVQDYVKGYFSNSIRMITIANEAIC-VGLNFEMVHH

3_2_Wolb_WP_208571653.1 -AESDYHGFLCGFLV-NFRYRA----VADFYPELLIG-----KGYADVVLLVRGVD-----QANDSVPIIIELKVGD-------------EEGLEQAKDYAKSCSVSSLPIHTSSPSAVC-VALNFQLRGD

3_2_Wolb_MBR9983825.1 -AESDYHGFLYGFLV-NFRYRA----MADFYPELLIG-----KGYADVVLLVRGVD-----QANDSVPIIIELKVGD-------------EEGLEQAKDYAKSCSVSSLPIHTSSPSAVC-VALNFQLRGD

3_2_Wolb_WP_164224553.1 -AESDYHGFLCGFLV-NFRYRA----VADFYPELLIG-----KGYADVVLLVRGVD-----QTNDSVPIIIELKVGD-------------EEGLEQAKDYAKSCSVSSLPIHTSSPSAVC-VALNFQLRGG

3_2_Wolb_WP_226079617.1 -AESDYHGFLCGFLV-NFRYRA----VADFYPELLIG-----KGYADVVLLVRGVD-----QTNDSVPIIIELKVGD-------------EEGLEQAKDYAKSCSVSSLPIHTSSPSAVC-VALNFQLRGG

2_2_Wolb_WP_065095360.1 -SESDDHGFLSGFLV-NFRYRA----MADIYLELLIG-----RGYADITLLVRGQE-----KLNNSVPIIIELKAGQ----------EHAGQALEQARGYVRNCPISSVSAHTSSRNAVC-VGLNFNH-NA

2_2_Sixo_MBP1525747.1 -NESDDHGFLSGFLL-NFRYRA----MADIYLELLIG-----RGYADISLLVRGQE-----KLNNSVPIIIELKAGQ----------EHAGQALEQARGYVRNCPISSVSIHTSSRNAVC-VGLNFNH-NA

2_2_Wolb_WP_114517506.1 -NESDDHGFLSGFLL-NFRYRA----MADIYLELLIG-----RGYADISLLVRGQE-----KLNNSVPIIIELKAGQ----------EHAGQALEQARGYVRNCPISSVSIHTSSRNAVC-VGLNFNH-NA

2_2_Wolb_WP_222837172.1 GKESDYHGFLAGFFM-NFRYQH----IANIYLELFVG-----GGYADITFLVRGRE-----RLTDSVPIIIELKAGA-------TRDKYADRALEQAENYVRKCPISSISIHTSSENAVC-VGLNFDL-QQ

2_2_Wolb_WP_191111445.1 GRESDYHGFVSGVLM-HFRYRN----VANIYLELFVG-----GGYADITSIVRGTQ-----RLINSVPCVTELKAGR-------RADRNAGRALEQAGNYVNGCPVSSISIPTLSPRAVC-AGVNFDFGNP

2_2_Wolb_WP_155968908.1 GKESDYHGFLSGFLM-NFRYRH----TAGIYLELFVG-----GGYTDITFLIRGVD-----RLRDSVPIIIELKAGG----------ESADQALEQAENYVIRCPVSSISIHTSSGSAVC-VGLNFDLDED

X_4_Rick_WP_157752277.1 HSENDFKSVLYGMFLYNTL-------GVIPITEKSLG-----TGKADLILTFL----------DNHHPLLLE-K--------LYSNTNLGSHNDQQLKSYIRHS-----RVLTHKSHLDG-IILRFNNKAQ

X_4_Rick_WP_158309377.1 HSENDFKSVLYGMFLYNTL-------GVIPITEKSLG-----TGKADLILTFL----------DNHHPLLLE-K--------LYSNTNLGSHNDQQLKSYIRHS-----RVLTHKSHLDG-IILRFNNKAQ

X_4_Rick_WP_196795995.1 HSENDFKSVLYGMFLYNTL-------GVIPITEKSLG-----TGKADLILTFL----------DNHHPLLLE-K--------LYSNTNLGSHNDQQLKSYIRHS-----RVLTHKSHLDG-IILRFNNKAQ

X_4_Rick_WP_197064228.1 VEENDFKSVLYGMFLYNTL-------GVIPITEKSLG-----TGKADLILTFL----------DNHHPLLLE-K--------LYSNTNLGSHNDQQLKSYIRHS-----RVLTHKSHLDG-IILRFNNKAQ

X_4_Otsu_WP_011944936.1 TEERDFQAILHGLFSFKKF-ENT---TLRAISEVTFG-----TGKADLVLAS------------NRSILYFELALLK-----EHNVDTLLNKKKQQLDKYLVNA-----KVITNDKNANG-VVLIFDSKTG

X_4_Otsu_WP_064591374.1 TEERDFQAILHGLFSFKKFEENT---TLRAISEVTFG-----TGKADLVLAS------------NHSILYFELALLK-----EHNVDTLLNKKKQQLDKYLVNA-----KVITNDKNAKG-VVLIFDSKTG

X_4_Otsu_WP_012461321.1 TEERDFQAILHGLFSFKKFEENT---TLRAISEVTFG-----TGKADLVLAS------------NHSILYFELALLK-----EHNVDTLLNKKKKQLDKYLVNA-----KVITNDKNANG-VVLIFDSKTG

X_4_Otsu_WP_045912852.1 TEERDFQAILHGLFSFKKFEENT---TLRAISEVTFG-----TGKADLVLAS------------NHSILYFELALLK-----EHNVDTLLNKKKQQLDKYLVNA-----KVITNDKNANG-VVLIFDSKTG

9_4_CAga_RDH39957.1 QKEFSFQAMMQGITAGLKK-DSF---ELRAFAEPNYSA----QGRADLVITLASFDAQKK-LLAE-RVFVFEFKALVS----SHKLQVNAAFALQQAKKYTE-----NLKSLSNAKEAMV-MGLVMNSYAT

9_4_CRic_WP_071662257.1 KKEFVFQAVMQGMIAGLNQ-ETC---QVRTFIEPNYSA----QGRADLVATLPSFNAQKE-LLTE-SLFIFEFKVLVS----SSMISRSVNAALEQAEKYVD-----NLKSLSDARKVAL-MGLVMNSQAK

9_4_Rick_WP_024547315.1 TSEKDFQSVVQGLFMGQIFNQCK----VNTFAEATSG-----TGRVDIVLKFINKNN-----EEHGTPIIIELKYQQF-------SNALLEHVQVQLEQYRD-----TLKAMTDQEKVIL-VSMIFNLQGS

9_4_Rick_MCC8418595.1 KQEVDVQIIITGMLMDQILANGK---HLKTFAEITFG-----SGRGDLAIFIIDENF-----YED-RPIIIECKYG---------SNAIITDQQIQ--GYIK-----YLKHATEHKQAVG-ITLILDPSKE

9_4_Dpon_XP_019765667.1 NSESDFQAILHGTFF-GKHDQLK---TIKVLPEANPSK----SGRIDLLIC--RREYMGSDVPQDEYILIMELKYAKD----KKDSENKLNDANKQILKYIS-----NLKSITDSRAIIP-ITLVFNKAAN

9_4_Bcal_CAF0888058.1 NSESDFQSIIQGFFL-GFSSSTKGRDIIKVLPEVNPSK----EGRIDLVAK--KFTDNGV-HINEDMIFVFELKFS-E----RFDIDKKLMEAKEQVSKYKS-----NLKSVTDLRVFTP-IIAVYDKN--

9_4_Wolb_WP_236515199.1 RKEKDFQAIVQGLLMNGEIKGGK---EVRVYTESNISS----KGRADLALSFMHFK-DDG-SFEEGCPIVIELKYAKG----IKQVKKALISAKKQLEEKYN-----LIKSFTSKKTAKS-LAMVFNEEAK

9_4_Npil_GFS42335.1 RKEKDFQAIVQGLLMNEKTEDGK---EVRVYTESNVSP----KGRADLALSFTHFK-DDG-TFKEGCPIVIELKYAGG----IKQVKKALKIAKKQLKEKYN-----LIKSFTSKKTAQS-LAMVFNEKAK

9_4_Wolb_WP_114517194.1 KKEIDFQAIIQGLFINGETEDGK---EIRVYMESNAPA----QGRMDLALSFTQLSKNVC-SFIEDDPIIIELKYATS----TSNADKELKKAEKQMGEKYT-----ILRSFTNKKTAKH-VAMVFNIGAK

9_4_Wolb_WP_149168923.1 KREVDFQAIMQGLLINGKTQDGK---EIKVYVESNISS----LGKMDLALSFTYLQGDES-TIEEDEPIIIELKCANS------SVKKKLGHAEKQMSDKYK-----ILRSFTDKRTAKF-LAMVFNQSSR

9_4_Wolb_WP_230609175.1 KREVDFQAIMQGLLINGKTQDGK---EIKVYVESNISS----LGKMDLALSFTYLQGDES-TIEEDEPIIIELKCANS------SVKKKLGHAEKQMSDKYK-----ILRSFTDKRTAKF-LAMVFNQSSR

8_4_Wolb_WP_172758984.1 EKEAHTQAVFHGAFSHYSDIKL(5)NRALVLTEFQTGR----GKRIDMLIHGIKFVGHDS-NAKEYDPVGLELKGPRK----DTTADALRDEANKQITDEYKRG--VTYKTLTDGKKVAF-MGVVFDKGAN

8_4_Wolb_WP_180807982.1 EKEAHTQAVFHGAFSHYSDIKL(5)NRALVLTEFQTGR----GKRIDMLIHGIKFVGHDS-NAKEYDPVGLELKGPRK----DTTADALRDEANKQITDEYKRG--VTYKTLTDGKKVAF-MGVVFDKGAN

8_4_Wolb_WP_155968920.1 EKEAHTQAVFHGVFSHYSDIKL(5)SRALVLTEFQTGR----GKRIDMIIHGVKFADQAR-SAGEYDPVGLELKGPRE----GRTADALKDEANKQIADEYMKG--VTYKTLTDGKEVGF-IGVVFDKGAS

8_4_Wolb_WP_168456001.1 NEEAHFQGALHGVFSHYSDIRL(5)NRALVLTELQTGR----GKRIDMLVHGIKFASRDG-NAKEYIPVGLELKGPRK----GEKADALVKEADEQIRTEYKEG--VSYKTLTDGDKVAF-IGVAFDEKAE

8_4_Wolb_WP_226080818.1 EKEAHTQAVFHGVFSHYSDIKL(5)NRALVLTEFQTGR----GKRIDMLVHGIKFADQAS-SAKEYDPVGLELKGPRE----GKTADALVKEANDQINTEYVKG--VTYKTLTDGKEVGF-IGVVFDKSVS

8_4_Wolb_WP_236515007.1 EKEAHFQAVLHGLFSHYGDIKL(5)SRALVLTEFQTGR----GKRIDMLVHGIKFADRGS-NAKEYIPIGLELKGPRE----GKTADALKDEANKQISDEYTKG--VTYKTLTDGKEVDF-MGVVFDKEAS

8_4_Wolb_WP_143688758.1 EKEAHFQAVLHGAFSYYSDIKL(5)NRALVLTEFQTGR----GKRIDMVVHGIKFADQAS-SATEYDPVGLELKGPRE----GKTADALKDEANKQIADEYTKG--VTYKTLTDGKEVDF-IGVVFDKGAS

8_4_Wolb_WP_211908378.1 NKEAHFQGILNGVFSYYSDLKL(5)TRALVLTEFQTGR----GERIDMLVHGIKFVAQGG-NAEEYTPIGLELKASRQ----GKRAQALLREANDQINEEYKEG--VTYKTLTDGDEVKF-IGVVFDKGSN

8_4_Wolb_WP_208571570.1 NKEAHFQGILNGVFSYYSDLKL(5)TRALVLTEFQTGR----GERIDMLVHGIKFVAQGG-NAEEYTPIGLELKASRQ----GKGAQALLREANDQINEEYKEG--VTYKTLTDGDEVKF-IGVVFDKGSN

8_4_CMlo_GHM58178.1 NKEAHFQGILHGVFSYYSDLKL(5)TRALVLTEFQTGR----GERIDMLVHGIKFAAQGR-NAEEYTPIGLELKASRQ----GKGAQALVREANDQINEEYKEG--VTYKTLTDGDEVKF-IGVVFDKGSN

8_4_Wolb_WP_179947478.1 NKEAHFQGILHGVFSYYSDLKL(5)TRALVLTEFQTGR----GERIDMLVHGIKFVAQGG-NAEEYTPIGLELKTSRQ----GKGAQALSREANNQIDEKYKKG--VTYKTLTDGDEVKF-IGVVFDKGSK

8_4_Wolb_WP_114517855.1 NKEAHFQGILNGVFSYYSDLKL(5)TRALVLTEFQTGR----GERIDMLVHGIKFVAQGG-NAEEYTPIGLELKTSRQ----GKGAQALLGEADDQVGEYEKGV---TYKTLTDGDEVKF-IGVVFDKGSN

7_4_Rick_HJD64690.1 GRESEFQGLLEGIFKYYSDTSL(5)RRVLVLTEFQTGA----GGRIDMLMQAIGPSEQG---TKEYVPVGLELKYDDS(20)DKAKHVVEKLLQDQTERYSKGA---AIKSITDGNKVAI-MGIVFNARAD

7_4_Rick_UCM86429.1 NSEVEFQGILEGIFKYYSDAKL(5)RRELILTEFQIGG----GGRIDMLVQAIGASEQA---TKEYTPVGLELKYDNS(20)QKVKKVVSKLLDEQVERYAQGA---AIKSITDGNKVAM-MGLVFNAQAQ

6_4_Wolb_WP_141456778.1 TNEARLQAILNGLFSSYSDLKL(5)-KTVIIPEFQVGA----GGRVDMVIQGIGPSPQG---AKEYTPIALEFKLIGKNLNQDQLKQEVDKLTKEQNIRYAKGA---ALKAITDSDKMFF-MGVVVNVKAK

6_4_Wolb_WP_226079864.1 TNEARLQAVLNGLFSSYSDLKL(5)-KTVIIPEFQVGA----GGRVDMVIQGIGPSPQG---TKEYTPIALEFKLIDKNLNQNQLKQEVDKLTKEQNIRYAKGA---ALKAITDSDKMLF-MGVVVNAKAK

6_4_Wolb_WP_172758417.1 TNEARLQAVLNGLLSSYSDLKL(5)-KTLIIPEFQVGA----GGRVDMVIQGIGFSHQG---TKEYTPIALEFKLIDKNLNQDQLKQEVDKLTKEQNVRYAKGA---ALKAITDSDKMLF-MGVVVNVKAK

6_4_Wolb_WP_214303288.1 TNEARLQAILNGLFSSYSDLKL(5)-KTVIIPEFQVGA----GGRVDMVIQGIGPSPQG---AKEYTPIALEFKLIGKNLNQDQLKQEVDKLTKEQNIRYAKGA---ALKAITDSDKMLF-MGVVVNVKAK

6_4_Wolb_WP_213863501.1 TNEARLQAVLNGLFSSYSDLKL(5)-KTIIIPEFQVGA----GGRVDMVIQGIGPSPQG---TKEYTSIALEFKLIDKNLDKGGMKKEVDKLTKEQNVRYSKGA---ALKAITDSDKMFF-MGVVVNVKAQ

6_4_Wolb_WP_226080720.1 TNEARLQAVLNGLLSGYSDLKL(5)-KIVIIPEFQVGA----GGRVDMVIQGIGPSPQG---TKEYTPIALEFKLIDKNLNQDQLKQEVDKLTKEQNVRYAKGA---ALKTITDSDKMFF-MGVVVNVKAK

6_4_CMlo_GHM58127.1 TNEARLQAVLNGLFSSYSDLKL(11)KIVIIPEFQVGA----GGRVDMVIQGIGPSPHG---TKEYTPIALEFKLIDKNLNQDQLKQEVDKLTKEQNIRYAKGA---ALKTITDSDKMLF-MGVVVNVKAK

5_4_Rick_KHO02170.1 TNEAYFEAVLSGLLNSYSDIEF(5)-KINVRPEFQVGG----GERIDLFINVMSKNPAD-----SDVLIGLELKYGDGRATVDSMERTLEQAEKVQLRRYGISR---NIRAITEGANFFITLPVGFVSRAE

5_4_Rick_WP_081996388.1 TNEAYFEAVLSGLLNSYSDIEF(5)-KINVRPEFQVGG----GERIDLFINVMSKNPAD-----SDVLIGLELKYGDGRATVDSMERTLEQAEKVQLRRYGISR---NIRAITEGANFFITLPVGFVSRAE

5_4_Rick_MCC8369105.1 TSEAYFAAVLSGLLNSYSDIEF(5)-KINVRPEFQVGG----GERIDLFINVMSKNPAD-----SDVLIGLELKYGDGRATVDSMERTLEKAEKVQLRRYGISR---NIRAITEGANFFITLPVGFVSRAD

4_4_Wolb_pdb|7ET0|A SNEATFQAVLHGLFSSYGED------NIKVITEFQIGG----GEKLDVMLVINATDQ-----KKEYPPVGIELKFAKK-----GELDKKEKDAKDQLKRYKEGE---AYKVITDAGKVKL-IYAVFNKGAT

4_4_Wolb_WP_006012795.1 SNEATFQAVLHGLFSSYGED------NIKVITEFQIGG----GEKLDVMLVINATDQ-----KKEYPPVGIELKFAKK-----GELDKKEKDAKDQLKRYKEGE---AYKVITDAGKVKL-IYAVFNKGAT

4_4_Wolb_WP_182319426.1 NNEATFQAVLHGLFSSYGDD------NIKVITEFQIGG----GKKLDIMLVINATDE-----KEECPPVGIELKFAKK-----GELDKKEVEAKGQLTRYKEGE---AYKVITDAGKVKL-IYAVFNKDAT

4_4_Wolb_WP_182365641.1 NNEATFQAVLHGLFSSYGDD------NIKVITEFQIGG----GKKLDIMLVINATDE-----KEECPPVGIELKFAKK-----GELDKKEVEAKGQLTRYKEGE---AYKVITDAGKVKL-IYAVFNKDAT

3_4_Wolb_WP_208571653.1 SREAKFQALLRGIFQSCDN-------PAKVIIEFQLQR----GRKIDLVLSKSAEN-------DDTHPIGIELKYANT----AEQVERKRVEANRQLSEYEFCG---GCKRITGGDAMVL-LYAILNAGQV

3_4_Wolb_MBR9983825.1 SREAKFQALLRGIFQSCDN-------PAKVIIEFQLQR----GRKIDLVLSKSAEN-------DDTHPIGIELKYANT----AEQVERKRVEANRQLSEYEFCG---GCKRITGGDAMVL-LYAILNAGQV

3_4_Wolb_WP_164224553.1 SREAKFQALLRGIFQSCDN-------PAKVIIEFQLQR----GRKIDLVLSKSAEN-------DDTHPIGIELKYANT----AEQVERKRVEANRQLSEYEFCG---GCKRITGGDAMVL-LYAILNAGQV

3_4_Wolb_WP_226079617.1 SREAKFQALLRGIFQSCDN-------PAKVIIEFQLQR----GRKIDLVLSKSAEN-------DDTHPIGIELKYANT----AEQVERKRVEANRQLSEYEFCG---GCKRITGGDAMVL-LYAILNAGQV

2_4_Wolb_WP_065095360.1 TSEAEFQAVLHGLFYTLGN-------PARVIIEFQLER----GGRIDLVLSRFVER-------DDTHPIGIELKFART----AGQIRQKSAEANRQLQRYTQCG---GCERITDGDQMVL-SYAVFNNGAR

2_4_Sixo_MBP1525747.1 TSEAEFQAVLHGLFYTLGN-------PARVVIEFQLER----GGRIDLVLSRFVER-------DDTHPIGIELKFART----AGQVRQRSAEANRQLQRYTQCG---GCERITDGDQMVL-SYAVFNNGAR

2_4_Wolb_WP_114517506.1 TNEHEFQAFLHGIFYALGN-------PAKVIIEFQLER----GRRIDLVLSRSVER-------VDTHPIGIELKFANT----GGQVQQRMAEANQQLQEYAQCR---GCVRVTDGDRMVL-SGVVLNDGAQ

2_4_Wolb_WP_222837172.1 NSEARFHSILHGIFYACDN-------PAKVLIEYQVGR----GRKLDLLLLRPIEHG------QNTHPIGEELKFARN----EGEVQQRMNEGEEQSAGYMSQR---GYKRVTDGNEIVL-SYAVFNNDAE

2_4_Wolb_WP_191111445.1 AREAGFHAALHGLFYTCDN-------PARVVSEFQVGG----GGKLDLVLSRAIGRM------GGTHPIGTELKFAAT----EADVQNREEEADEQVEGYLQSR---GFDRITDGDKMVF-SYAVFNDQAP

2_4_Wolb_WP_155968908.1 AREVGFHSVLHGLFYTCDN-------PARIIIEFQLGG----GEKIDLVLLRSAESR------GGVHPIAKELKFA-D----DYELQSKIQEANNQLNSYLQCR---GYKRITDGDTVVL-SYAIWNDRAQ

CR7_bact_KKQ32643.1 QKEAYYHSLFQLIGTLL---------GFDIQSEVLTS-----VGRIDLLLQT------------SKHIYIFEFKFG-----------KTVEEAMAQIKDRRYYE-----KFLNYGKEIFL-IGMVFDFKDK

CR7_bact_KKR95525.1 RGEAFYQTIFFLICQAA---------GFKTRVEVATA-----LGSIDAMIEF------------GSKKYIFEFKID-----------DSAKKALEQIKRKEYYR---GFLAQATKQQLVL-VGVSFDTVTR

CR7_bact_HDT11365.1 KQEAYYQSIIYLTLKFL---------GVYIDCEVSTN-----RGRIDGVIKT------------DKYIYIMEFKMA----------PATPQDALNQIKEKGYHI-----SYQTDNREKYI-IGISFDPEKK

CR7_bact_MBU1754308.1 KYEGYYASIFYAVFASL---------GYDLEVEESTN-----RGRIDMAIKT------------DKVVIIFEFKVV--------ERESGQNTALEQIKDRKYYE-----KYLTSGKGVYL-VGIEFSKAER

CR7_Vbac_MBS0627173.1 EKEYFYHGLLQMAFSSA---------GIKCQSEYSTS-----HARIDLILEL------------QNKIYIIEVKFN-----------ESAEVALSQIEARRYYE-----PFLNSGKAITL-LGLSFIKKPN

CR7_Tcas_EFA06969.2 TQSSRPVVKVSEQLRDAKT------SSQNKRREKHGR-----KNECDICVTR------------GTGGAILELKLLN-Q----INANENSKAALQQIIDGKYHT-LFDRDEYKHVRTKIY-VGLCFKKNL-

CR7_Taer_AHF01847.1 NYEGFYASVIYVYLQSL---------GLNIIGEDVTN-----QGRIDLTVIM------------DHAIYIMEFKVDHPD----KPSSQSSTNALQQIKDKRYVD-----KYLAVKKPIYL-IGIHFNAEQK

CR7_Tdis_HFC98578.1 ADERFFHTVFYLALALS---------GYRAESEVLTH-----RGRLDMAVRYEG----------ENRVFVFEFKAG-----------ISAEEALKQIEARGYAD-----RFRSRGLSVIS-VGVSFDPAER

CR7_Tmol_CAH1377499.1 HDELKAVLFTLCVNEFD----------PSCERFCQDNK----KNRLDLLFIRK-----------DGTGIIIEVKFG----------QSSAQAGLKQIFDKEYYK---VFEGENIIKDKIY-IGLHTNHQ--

CR7_Tmol_CAH1381491.1 HFEIQADLYFLIKDWID----------ECCYAELKVDSGN--SPRIDIFFKIN-----------DNTSVVLEIKHFIS--------AKSVDEALEQILDIKYYK--VCSNCNITTENKIL-LGVFLASDG-

CR7_Ssp._HAK29896.1 EKEGYHHSNVHLILKML---------GFDVISELSTN-----TGRIDMCLQL------------IDKIYIMEFKFN--------EELDSSEEALQQILDNKYWE-----RFITDSKKIIC-VGIGFCGKGR

CR7_Ssp._MCI6100144.1 KAEKYYQSIIYTLCRLC---------GMDILTEVATN-----KGRIDAVLDA------------GKHLYILEFKLQ-----------KTADAALAQIDEKSYAE-KYLRPAREKGQTIHA-VGINFSYAPE

CR7_Sbac_MBL7791055.1 TEESYFHSVTHVVLALT---------GHVVHSEIPTN-----QGRMDAVLDA------------GERLYIFEFKLR-----------ESADAALAQIHERGYPE-----RFRAAGKQLTL-VGVAFDVEAK

CR7_Sbac_MBF0239753.1 EKERYYQSIFYVIFALL---------GVHVKAEERTS-----TGRIDAVIET------------ENYIFIFEFKMASGD----P--DKICDTALQQIREHEYFV---KYAQPGQQKNILL-IGTAFDLDER

CR7_Rcal_WP_250630933.1 RYEAFYHAIFFMAFSLM---------QTYTQTEVQTA-----KGRIDVVVYT------------PDAFFVIEFKV-----------NGSAAEALAQIKAKGYHQ-----KYLHQNREVVL-IGLGCADKEV

CR7_Rbac_MBX9924192.1 NDEKFYHALLQTIFSAA---------GIEVQSERITS-----IGRMDLILEL------------PNVIYILELKMN-----------ESPEIGIDQIEKQKYYE-----PFLGKDRKILA-VGMSFSRKKQ

CR7_Rsp._MCS6794414.1 KKEAYYHSVLFLALKLA---------GFHVQAEVRTS-----KGRIDAILQY------------DNRTYLFEFKLD-----------QNAQIALQQIEQKEYFK---KYLQQPNQHKIYL-VAINFSSEKK

CR7_Pbac_NBV99705.1 KKEKYYQGILYTILYLT---------GLKINSEEPTS-----TGRIDLVLKT------------DSEIYIFELKLN-----------QTAKAALEQIHKKKYHE-----KYLSLNTKITL-IGLNFNSEIK

CR7_Psp._MCO6480960.1 KTEAAYHSVLYTALSLL---------GFHIRCEVATG-----EGYADAVIET------------GTHIYIMEFKVG-----------RSPQEALNQVRERRYAK-----AFQADEREGLL-VGVSFSTEFK

CR7_Psp._WP_053405472.1 KQEGYYAAVAYTYFRSI---------GFRINAEEASA-----QGRADLVIDT------------GQSIYVIEFKVD---------AKNP-GEALRQIKDRGYHE-----PYLGQGLSVYL-IGIHFNSEQK

CR7_Ptep_XP_042902046.1 QDELKGIMFAFAFKDDM---------FSKVESECHVVPER--RFRADLVIIKK-----------DGIGVTVEIASG----------NDSEEEALQKIFDKQYFK-----ALPDSIEQKIY-IGLQFNYSIG

CR7_Pbac_MCQ2218441.1 LIESNFRNLLYLMIRAT---------GYNVTVERPVL-----SGRIDLFFET------------NNCIYIIECKRD-----------ESAEAALSQIDEKRYAE-----KVSSHDKKIVK-IGANFSTKER

CR7_Mbac_MBQ9465894.1 KTEKDYETILRVLFDGL---------GIKVDTEVKNA-----TGRCDVVMKS------------KTTVYVLELKLDG---------NGTVDDALAQIDEKNYLI-----PYWNDPRPKVK-VGVLVDPTRR

CR7_Mbac_MCL2091372.1 RFEGYYASVFYACFASL---------PVDVTTEDASS-----TGRLDMAVHV------------ADRTYLFEFKTV---------DHSPTGKALQQIRDKRYAD-----KYRDDGTTIHL-VGIEFSQEQR

CR7_Mbac_TSA57635.1 PLESYYHSLLQMICTTA---------NIKTQSEYNTS-----HGTIDFVMDL------------PKVLYVVEVKFN-----------KTAQLALDQIKTKRYYE-----PFTDQNKPIIL-LGLNFAKSAG

CR7_Lmuc_WP_022952435.1 EYEGYYASVFYSYFASL---------GLDVTVEDATN-----LGRIDMTLKF------------NQQIYIFEFKVV---------DDEAKGEALQQIKDKAYAD-----KFRAPDRPIYL-IGVEFSKKTR

CR7_Kbac_MBR0066306.1 QNEQTWQAIMYVVLRVV---------GINVGGEVRTH-----KGRIDLTVET------------ATDAYIIEVKRD-----------STAKKAIEQIQDKSYTD-----RFRPSGKPITL-IGLAFSTKKR

CR7_Kbac_MBQ6011453.1 -AEAKRYFLLFSAM--L---------GANPAPEFPSV-----RGYADAVIET------------PSAVYVFEFKYG-----------KSAKAAIRQIRARGYAD-----KWIGGKRPVTL-IGINFNPRKR

CR7_Ibac_MCK6605618.1 DEEKYYHFLFIMIMYMS---------GIEIESEVNTN-----IGRIDGVIET------------KDKIYIIEFKLD-----------KTPGEALQQIFRKKYYE-----KYTGREKEIVL-MGVSFTCGDI

CR7_Dmex_WP_139447449.1 GYEGYYASIFYCYFASI---------GLDVRAEDTTN-----QGRLDMSVKF------------EDRVYVFEFKVK--------RLSQNNKSALGQIKDKKYYE-----KFISDFKDIYL-IGVEFDSETR

CR7_Dalk_WP_144686569.1 ACEGHYASVFYSYFASM---------GVDVTPEDRTN-----RGSMDLSLRT------------DDKVYIIEFKMD--------SQRKNAAAALQQIKDKGYHE-----KYRAQGKKIYL-VGMVFSAEER

CR7_Dbac_HDI78790.1 IKEYYYHTIMYLSLSLL---------GYDARTELLSS-----KGRLDMAVIF------------KDRVYVLEFKVG-----------KGAEDAISQIREKGYAD-----RFIAEGKKVIL-CGISFDDKSR

CR7_Dcel_WP_146889543.1 RKEAFFNSLMHLMLMST---------GLPTLSQVQTS-----KGRMDTVIEL------------EKQVFIFEFKLD-----------GTAQEALEQIKGAGYGE-----KYL--GKRVVM-VGVNFDSASK

CR7_Cbac_TAG00760.1 RKEAYFHTIFHLTFNMI---------GFRCESQVQNN-----IGRIDTIVET------------ELYIYIFEFKVD----------TKKDNFALEQIKNNQYAN-----KYFALEKEIFA-FGINFSSEKR

CR7_Ctan_PWM21203.1 RDEKGFQLKFYLIFRLL---------GIQIDSEVKTE-----TGRVDAVVQH------------QGSTYVMEFKYDG-----------SAGEALRQIDERGYLI-----PYEALDSRLVK-VGVNFSSKEQ

CR7_Csep_XP_044763649.1 QNDFELQALLYHFVKKY---------FPSLKGEALTMPKR--YLKCDLGLPID-----------DKVGVIIEITTYD---------RSTSLDALSQIVANKYNE----ILSFEGLTDEVY-IGLHLGED-R

CR7_CSfa_HIV09552.1 VPEAAYQRILYVLLASW--------RFFRVTAEDRQA-----QGRADLVAES------------RRAVYVFELKIK-----------GTAQEALAQIKDRGYAE-----PYRATGKPIHL-IGLAFDPSNH

CR7_CNve_MBF5058916.1 AREGFYHAVFLSLLEGM---------GIKTYSEQKTN-----IGRIDLVVEM------------PLIIYIFEFKKD-----------QSADAALEQIKNTKYGE-----KYTQSGKKLIH-VGVNFSSKSR

CR7_CDba_MBA2307071.1 HHENFYHTMVQSFFILL---------GFETESEISTN-----RGRIDLAVFT------------KKFTYIFEFKVR-----------TSPETALKQIEDRAYYERYLSSPKIKKESTVVL-VGMTFSKRYG

CR7_CDba_MBS1988097.1 PDESFYHTLFHMITTVI---------GFATSSEDSTH-----TGKPDLIVQT------------KDRIYVIEFKFN-----------KSTGQGMQQILDRRYYE-----RYRDLGKQIVL-VEITFMYQNK

CR7_CDba_MBA3751962.1 NQEAYYHSLFQLLVIML---------GYESQSELTTN-----KD-IDLVITT------------KNHIYIFEIKLK-----------GTPAAALKQIQKRGYSE-----RFALSGKKITV-VGIAFNPLQG

CR7_CDba_MBM3893910.1 PQENLYQLVFHYLFFAS---------NLRTTMEETTN-----RGRIDIVVET------------VRAVFLLEIKMN-----------EPASKALQQIKDMDYAA-----KYRHQKQEIYA-VGICLSSKER

CR7_CDba_MBM3893379.1 ARENMYQIIFTILFTAC---------GVRVTTEEHTN-----RGRIDMVLEV------------EDMVYVVELKMD-----------QPAAVAMAQIKEKEYSA-----KFKHTGKQLYA-VGIAVNTETR

CR7_CChe_WP_243018173.1 KTEKDYHSNLHMLLYGLNCLDG---INSHVASESPSS-----KGMADIVLTI------------GSIVYIIEIKYK-----------ANGKVALQQIKENRYYT-----SYLWKAKEIIL-LGLNFDQETK

CR7_CBma_WP_023792718.1 LKEKDYHTLIQFILSVL---------SINAQSETITN-----AGRIDLILET------------KNIIYIFEFKVN-----------QSAEIALQQIKDKKYYQ-----RFLDKNKKIIL-VGLNFISSKD

CR7_Cbac_MCB9493532.1 STEKHYQTIFYVTCLVA---------GFEPIAEDHTA-----RGRIDVTFEH------------EHVIYIIELKIG-----------CSADNALAQIHEKRYYE-----KFLNKGKRIVL-LGISFDVQAK

CR7_Cbac_MCB0121024.1 PQEAYYHSVVYLVLKLL---------GFTIHAEWPTN-----LGRIDAVLEL------------PDTIYLLEFKMS-----------SGA-IALQQIRDKQYAQ-----PFLGSDKTVIL-LGIAFDRESR

CR7_Bsp._WP_026669493.1 KYEKYYQSLIYLACRMS---------GMEIISENRTN-----IGRIDATLTA------------GKYIYVIEYKID-----------KSAKEALEQIEDKKYTE--KYVDKLKNGFNLIK-VGINFSSSKD

CR7_Bbac_MCS6967662.1 RYESFYHAIFYLAFSML---------GVYALSEVHTS-----RGRIDVVVEI------------GRWVYVIEFKVG----------EGGGEEALRQIKERGYYQ-----RYMHRGKEVVL-MGLGCYQKQV

CR7_Bbac_TAE16172.1 DKEAYYHAIVFISLQIV---------GLNVQAEVSQA-----KGRADAVIHL------------DNDIYILEFKLD-----------ESAEIALQQIKDKGYAT-----PYLQAGKNIYL-LGLNMTSKDK

CR7_Bbac_TAE15043.1 NREKFYHAIVYLVFRLL---------GYFTQIELNSG-----RGRLDCVVFW------------GNKVFLFEFKLD-----------KSAEEAIAQIKAKNYYA-----PYQGVGKDIYL-IGVNMTSAQK

CR7_Bbac_MBU0763457.1 PKENYFHSLFYLVVKLL---------GYIIESEIQTS-----DGRIDAVVWT------------DNFRYIIEFKM------------GKAKEAMQQISEKLYHL-----KYTTDIKKIFL-LGIGFDAVKK

CR7_Bbac_TAE69485.1 KKESYYHAIIFLTFKLL---------GYMVEAEVRTS-----KGRIDAVVNY------------DNVVFLFEFKVN----------ASTSQTALNQIFEREYFQ-----KYLQPNKEIYL-IGMNCYEKEI

CR7_Bbac_NJO02823.1 DREAYYHAVTYLALSLM---------RSFIRAEVSQS-----KGKPDAVVFT------------DTTIYVMEFKMD-----------DSAEAAMKQIREKAYVK-----PYLKLGKPVKA-VGIHFNSGQK

CR7_Bbac_GHT02692.1 SNEKEYQYVFYVLMTLL---------GEFTFAEVRGN-----IGRADAIIKT------------DDTVYLFEFKVTS---------AGTVDEALAQIEAKGYAK--PFETDPNEKRKIVK-IGVVFDTATR

CR7_Bbac_GHT52288.1 ANEALYHIIFQLWMTML---------GFNIQSERMTN-----RGRIDAVLQQ------------EGVAIVVELKYH-AT----TKLDTLLNQAIAQIRENRYYE-----PFL--DRKIIL-LGIAFSGKDV

CR7_Bbac_MBL7979730.1 DQERYYHSVLFIIFQLL---------SVNTQVEVQVA-----GGRSDMVVQT------------KSHTYVLEFKLN-----------ENAVAALSQIHSKGYAT-----PYLHQGREVVA-VGINFSGAEK

CR7_Bsp._MBD5292592.1 KREVYFQNNLFIILSLV---------GLECREEVQTS-----YSRMDLFMRT------------TNYIYVIEVKLN-----------RTARQALEQIKKKEYDL-----PFERHNLPIVR-LGINFSTRTR

CR7_Bsp._MBD5310553.1 MPEIYYENNLYILFTII---------GIRTSVEVETS-----DGRMDVLMEC------------RQHIYVIELKLD-----------RSPEEALEQIRRKHYTL-----SLERHGIPVTL-IGINFSTETR

CR7_Bbac_MBR0301437.1 KREAYFKRLFYVVFSFM---------NIQIYTEVMNS-----EGRTDAVMVL------------NDTVYVIEFKLDA---------T-SAKEAIEQIDTRGYTA-----RFAASNRKVRK-LGLNFSSRTG

CR7_Bbac_MCI5981814.1 YTEALFQNAFYILFTLL---------GYDVRSEVRFA-----HGRADAIVQT------------SDTVYIFEFKRDG-----------SATEAIEQIRNQAYDV-----PFRALNKRIRL-VGVNFSSQTK

CR7_Bbac_MCQ2975740.1 KCERFYQNIIFMMFSMF---------NKNTHVEVKSI-----RGRADIVVFG------------KKSVFVLEIKIN-----------STPEEALQQIDDKGYSI---AYQDRINGRKVVK-CGVNISSKER

CR7_Bbac_MCI5777693.1 KNEAHYHDVFMAACGAM---------GFEVRAETPSS-----HGVSDLELFT------------KRTIYIFEFKVD-----------STPEDALRQIETQDYAL-----RYSHDTRTVML-IGVNFSSSTR

CR7_Bbac_MCF0188887.1 KGEHTYHAIFYTLFSLL---------GFAPKIENGCA-----RGYSDMIFET------------PTSVYCMEFKVD-----------RSAAEALRQIEERGYLE-----PFVGKGKRLVM-VGVNFSTKER

CR7_Abac_MCU0285016.1 TREAYYHSLIYIALQLS---------VIDVMAEIQTA-----LGRSDIVVSL------------EHYTYIIEFKMG------------SAQDALDQIEKKQYYA-----PYLGKNKTIIL-LGIGLSEKER

CR7_Aped_WP_279382584.1 PYERYYQSLFHMLFVLL---------GLEIHAEVPTN-----RGRIDHLLKT------------EQHIFIFEMKFL-----------RAASEAIQQIKDKRYPS-----GFASDGRQVVL-VGMSFEDSGL

CR7_Vbac_MBP5672899.1 VQEYAFERNLLVLLWAL---------NIRCDKEVRQS-----NGQADIVAVS------------GVGVFIFELKVD-----------ESADEALKQVKDKHYDA-----PYQGRNLPIWL-IGLNFDRKTR

Consensus/80% .pE..bpshbhshb.............h.h..E..ss.......bhDbhl..............p.h.bhbEbKh...............ppsbpQhppb...........bspspphhh.lslsbs....

**HHH** 🡪 Alpha helix

**EEE** 🡪 Beta strand

**Supplementary Figure 4. Structural analysis of CifB REase2 domain (left) & CR-REase-7 domain (right).**


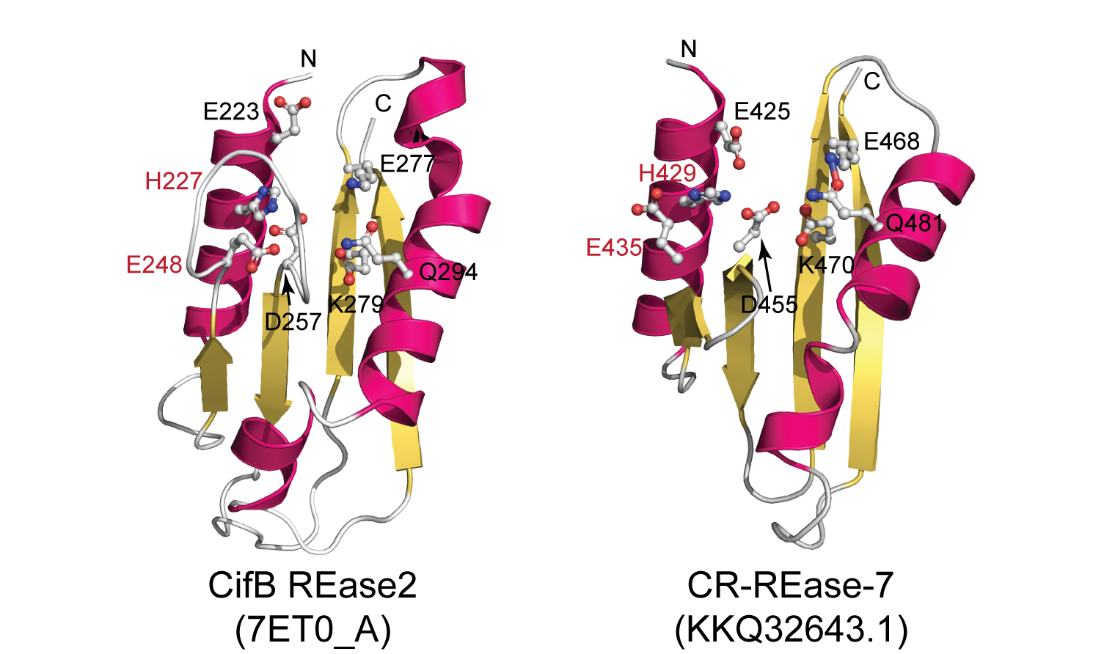

Supplement: evae171_Supplementary_Data [file evae171_supplementary_data.zip › SupplementaryFile1234.docx]
